# Deep learning radiomics-based prediction model of metachronous distant metastasis following curative resection for retroperitoneal leiomyosarcoma: a bicentric study

**DOI:** 10.1186/s40644-024-00697-5

**Published:** 2024-04-16

**Authors:** Zhen Tian, Yifan Cheng, Shuai Zhao, Ruiqi Li, Jiajie Zhou, Qiannan Sun, Daorong Wang

**Affiliations:** 1 Northern Jiangsu People’s Hospital, Clinical Teaching Hospital of Medical School, Nanjing University, Yangzhou, China; 2 Department of General Surgery, Northern Jiangsu People’s Hospital, Yangzhou, China; 3 General Surgery Institute of Yangzhou, Yangzhou University, Yangzhou, China; 4 Yangzhou Key Laboratory of Basic and Clinical Transformation of Digestive and Metabolic Diseases, Yangzhou, China

**Keywords:** Retroperitoneal leiomyosarcoma, Distant metastasis, Deep learning, Radiomics

## Abstract

**Background:**

Combining conventional radiomics models with deep learning features can result in superior performance in predicting the prognosis of patients with tumors; however, this approach has never been evaluated for the prediction of metachronous distant metastasis (MDM) among patients with retroperitoneal leiomyosarcoma (RLS). Thus, the purpose of this study was to develop and validate a preoperative contrast-enhanced computed tomography (CECT)-based deep learning radiomics model for predicting the occurrence of MDM in patients with RLS undergoing complete surgical resection.

**Methods:**

A total of 179 patients who had undergone surgery for the treatment of histologically confirmed RLS were retrospectively recruited from two tertiary sarcoma centers. Semantic segmentation features derived from a convolutional neural network deep learning model as well as conventional hand-crafted radiomics features were extracted from preoperative three-phase CECT images to quantify the sarcoma phenotypes. A conventional radiomics signature (RS) and a deep learning radiomics signature (DLRS) that incorporated hand-crafted radiomics and deep learning features were developed to predict the risk of MDM. Additionally, a deep learning radiomics nomogram (DLRN) was established to evaluate the incremental prognostic significance of the DLRS in combination with clinico-radiological predictors.

**Results:**

The comparison of the area under the curve (AUC) values in the external validation set, as determined by the DeLong test, demonstrated that the integrated DLRN, DLRS, and RS models all exhibited superior predictive performance compared with that of the clinical model (AUC 0.786 [95% confidence interval 0.649–0.923] vs. 0.822 [0.692–0.952] vs. 0.733 [0.573–0.892] vs. 0.511 [0.359–0.662]; both *P* < 0.05). The decision curve analyses graphically indicated that utilizing the DLRN for risk stratification provided greater net benefits than those achieved using the DLRS, RS and clinical models. Good alignment with the calibration curve indicated that the DLRN also exhibited good performance.

**Conclusions:**

The novel CECT-based DLRN developed in this study demonstrated promising performance in the preoperative prediction of the risk of MDM following curative resection in patients with RLS. The DLRN, which outperformed the other three models, could provide valuable information for predicting surgical efficacy and tailoring individualized treatment plans in this patient population.

**Trial registration:**

: Not applicable.

**Supplementary Information:**

The online version contains supplementary material available at 10.1186/s40644-024-00697-5.

## Background

Retroperitoneal leiomyosarcoma (RLS) is a relatively common histologic subtype of retroperitoneal sarcomas (RPS) that is usually incurable upon the onset of metastasis [1–3]. RLS typically manifests as a large soft-tissue mass featuring areas of necrosis located within the perirenal or posterior pararenal spaces. The most frequent growth pattern, accounting for 65% of cases, is an entirely extravascular or extraluminal mass [4]. Moreover, the mass usually exhibits a well-circumscribed margin but can appear infiltrative on occasion. En bloc resection remains the cornerstone of treatment for RLS and is the only modality with curative potential [5–7]. Of the various subtypes of PRS, RLS is particularly recalcitrant and prone to distant metastases, with 55–78% of patients developing metachronous distant metastasis (MDM) within five years post-surgery, even after undergoing R0 resection [4, 7, 8]. Metastatic RLS accounts for approximately 75% of sarcoma-related deaths, with a median survival time of just 16 months and an overall 5-year survival rate of less than 50% [4, 7]. At the time of diagnosis, approximately 9% of patients will have already developed metastasis; however, most patients develop metastasis during the postoperative follow-up period. Thus, preoperative assessment of the risk of MDM is essential in this patient population to predict surgical efficacy and guide targeted intervention strategies. Studies have provided conclusive evidence that preoperative radiotherapy increases the rate of complete histological resection [9]. Adjuvant chemotherapy may also help eradicate undetectable micro-metastases that result from circulating tumor cells that are released into the bloodstream from primary or metastatic foci [10]. In addition, the establishment of dedicated sarcoma teams in tertiary care centers, along with the discovery and implementation of novel drugs and immunotherapeutic agents, have led to improved outcomes in patients undergoing sarcoma treatment. Despite the substantial progress made to date, several controversies persist; for example, differences in characteristics and uncertainties in the risk of MDM among the patients included in various studies may have contributed to the discrepant results reported in the literature. Therefore, a noninvasive tool to identify patients with RLS who have an elevated risk of developing MDM is urgently needed to guide clinical interventions and inform the design of clinical trials.

A prognostic nomogram that integrates clinicopathological variables could serve as a valuable tool for providing patient counseling, scheduling surveillance imaging, and determining eligibility for clinical trials. Pathological evaluation of surgical specimens can only be performed postoperatively; thus, radiological imaging continues to play a critical role in the diagnosis of suspected RLS [11, 12]. Contrast-enhanced computed tomography (CECT) is recommended by the National Comprehensive Cancer Network (NCCN) Clinical Practice Guidelines for monitoring the development of metastasis in patients with RLS [13]. Tumor-related ‘semantic’ features, such as tumor size, lymph node enlargement, and the involvement of adjacent tissues are well-established prognostic factors that impact survival outcomes [14, 15]. However, conventional visual assessment of the semantic features of lesions by radiologists may be inadequate in some cases in which there is a relative paucity of such features, and reliance on this technique alone may fail to capture a great deal of information about the spatial heterogeneity of tumors [16]. In addition, the heavy workload imposed by manual image evaluation can lead to fatigue among radiologists, increasing the likelihood of an overlooked lesion and ultimately leading to a decrease in sensitivity. Consequently, the search for novel reliable prognostic markers is warranted.

Radiomics offers a valuable approach for assessing tumor prognosis by enabling the extraction of mineable high-throughput quantitative features from medical images. This approach facilitates the capture of tissue features and lesion characteristics, such as the heterogeneity and shape of tumors, that are not discernible to the naked eye of radiologists [17, 18]. Deep learning, a form of machine learning involving convolutional neural networks (CNNs), has achieved impressive performance in the automated analysis of visual images. This advancement has accelerated the integration of radiomics into medical imaging methodologies and emerged as a new paradigm in personalized medicine [19, 20]. Accumulating evidence suggests that combining conventional radiomics models with deep learning features can yield superior performance in the assessment of tumor prognosis [21, 22]. However, no studies to date have evaluated the use of CECT-based radiomic models that combine deep learning and hand-crafted radiomic features to predict the occurrence of MDM in patients with RLS.

Thus, the aim of this study was to construct and validate a novel CECT-based deep learning radiomics nomogram (DLRN) for the preoperative prediction of MDM risk in patients with RLS. This model could facilitate the development of a personalized approach for targeted interventions and enable continual monitoring of high-risk individuals while simultaneously avoiding the over-monitoring and over-treatment of low-risk populations.

## Methods

### Patients

**Fig. 1:**
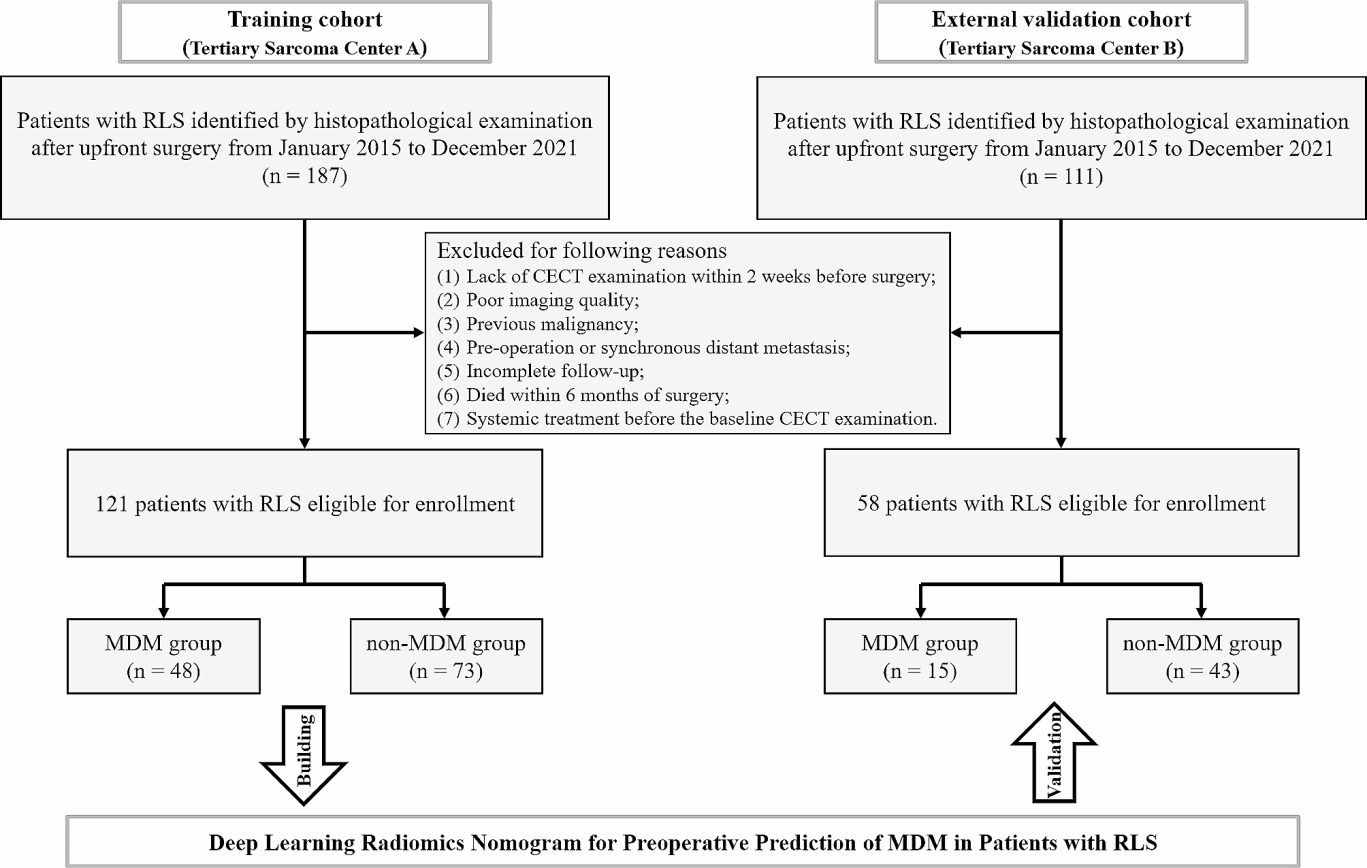
Flowchart of patient enrollment. DLR, deep learning radiomics; MDM, metachronous distant metastasis; CECT, contrast-enhanced computed tomography

Patients who had undergone surgery and achieved margin-negative (R0) resection and were subsequently diagnosed with histopathologically confirmed RLS were included in this study. An R0 resection was defined as the complete removal of macroscopic tumor with microscopically tumor-free resection margins [23]. The pathological diagnosis was based on the consensus of two pathologists who had evaluated the surgical specimens postoperatively. The exclusion criteria were as follows: (1) lack of abdominal CECT scans within the one-month period before surgery; (2) poor imaging quality; (3) the presence of synchronous distant metastasis (occurring at baseline or within six months after surgery); (4) previous malignancy or other coexisting malignant tumors; (5) treatment with anticancer therapy at or before the baseline CECT scans were conducted; (6) incomplete clinical indicator or follow-up data; and (7) death from non-metastatic causes within the 6 months following surgery. Based on these criteria, a total of 121 consecutive patients who were treated at a tertiary sarcoma center between January 1, 2016, and December 31, 2021 were included in the training cohort. This cohort comprised 60 men aged 60 [49, 65] years and 61 females aged 58.2 [48, 67] years. Additionally, to assess the reliability of the model, an external validation cohort comprising 58 patients who received treatment from another tertiary sarcoma center, including 29 male and female patients aged 61 [46.5, 66] and 59 [43, 67.5] years, respectively, was established. A flow diagram of patient enrollment is shown in Fig. 1.

At our center, retroperitoneal sarcomas are treated by an experienced multidisciplinary team of specialists throughout the entire process. The dedicated sarcoma team includes radiologists, medical oncologists, radiotherapists, anesthesiologists, surgeons, pathologists, nutritionists and specialist nurses so that every decision is supported by knowledge of the latest scientific evidence and available clinical trials.

### Image acquisition

Arterial, venous, and delayed-phase CECT images were retrieved from the Picture Archiving and Communication System for further evaluation. All CECT images were reviewed independently by two professional radiologists with more than 15 years experience in abdominal imaging diagnosis. Cohen’s Kappa coefficient and intraclass correlation coefficient (ICC) were used to evaluate inter-observer agreement for semantic features (Supplementary Appendix 1) [24]. In this study, the Cohen’s Kappa coefficients/ICCs for semantic features showed good agreement between the radiologists as detailed in Table S1. Data related to the semantic features of the tumors obtained from the CECT images are presented in Table 1.The clinical tumor, node, metastasis (TNM) staging was based on the 8th edition of the American Joint Committee on Cancer (AJCC) Staging System [25]. The models and parameters of the CT scanners are provided in Appendix 1 of the Supplementary Materials.

**Table 1:** Clinical characteristics of patients in the training and external validation cohorts

Variable	Training Cohort (*n* = 121)			External Validation Cohort (*n* = 58)			Training vs. Validation
	non-MDM (*N* = 73)	MDM (*N* = 48)	*P* value	non-MDM (*N* = 43)	MDM (*N* = 15)	*P* value	*P* value
Age (years, median [IQR])	59 [50, 64]	60 [48.2, 66.3]	0.693	56.0 [47.5, 62]	66.0 [38.5,69.5]	0.282	0.81
Gender (n, %)			0.158			0.764	0.959
Male	33 (45.2)	28 (58.3)		21 (48.8)	8 (53.3)		
Female	40 (54.8)	20 (41.7)		22 (51.2)	7 (46.7)		
*Ki-67* index (%, median [IQR])	10 [5, 30]	20 [5, 40]	0.454	10.0 [5, 30]	10.0 [5, 40]	0.596	0.43
Tumor size (n, %)			< 0.001			0.885	0.231
≤ 10 cm	41 (56.2)	6 (12.5)		21 (48.8)	7 (46.7)		
> 10 cm	32 (43.8)	42 (87.5)		22 (51.2)	8 (53.3)		
Clinical N stage (n, %)			0.455			0.272	0.975
N0	63 (86.3)	39 (81.2)		35 (81.4)	14 (93.3)		
N1	10 (13.7)	9 (18.8)		8 (18.6)	1 (6.7)		
Cystic spaces or necrosis (n, %)			0.338			0.364	0.36
Not Present	34 (46.6)	23 (48.0)		20 (46.6)	10 (66.7)		
Present	39 (53.4)	25 (52.0)		23 (53.4)	5 (33.3)		
Degree of enhancement (n, %)			0.332			0.642	0.683
Higher than muscle	64 (87.7)	39 (81.2)		35 (81.4)	13 (86.7)		
Slightly below or equal to muscle	9 (12.3)	9 (18.8)		8 (18.6)	2 (13.3)		
Enhancement pattern (n, %)			0.037			0.587	0.112
Heterogeneous	63 (86.3)	34 (70.8)		38 (88.4)	14 (93.3)		
Homogeneous	10 (13.7)	14 (29.2)		5 (11.6)	1 (6.7)		
Tumor contours (n, %)			0.712			0.07	0.862
Smooth	39 (53.4)	24 (50)		26 (60.5)	5 (33.3)		
Irregular	34 (46.6)	24 (50)		17 (39.5)	10 (66.7)		
Adjacent organ involvement (n, %)			0.224			0.16	0.903
Not Present	60 (82.2)	35 (72.9)		36 (83.7)	10 (66.7)		
Present	13 (17.8)	13 (27.1)		7 (16.3)	5 (33.3)		

Note: The tumor size and N stage was referred to the 8th edition of the American Joint Committee on Cancer (AJCC) staging system

Abbreviations: MDM, metachronous distant metastasis; IQR, interquartile

### Region of interest (ROI) segmentation

Each sarcoma ROI was manually delineated at the cross-section of the primary lesion by radiologist 1 using the ITK-SNAP software (Version 3.8, www.itksnap.org) based on CECT images with a 5-mm thickness. Contouring was carefully performed within the borders of the tumors on arterial, venous, and delayed-phase images, avoiding covering the adjacent organs and tissues. Figure S1 and S2 show an example of the manual delineation method. Three weeks later, 60 patients were randomly selected, and the delineation was repeated by radiologists 1 and 2 to calculate interclass and intraclass correlation coefficients (ICCs). An ICC > 0.8 was considered to be indicative of good reliability and reproducibility. Detailed explanations can be found in Supplementary Appendix 2.

### Image normalization

The Combat compensation method (https://github.com/Jfortin1/ComBatHarmonization) was used to retain outperforming features in texture patterns. This method filtered the radiomic feature values affected by inconsistencies in imaging protocols, scanners, and parameters, thus improving the sensitivity of data acquired on different CT equipment [26, 27]. This method has been widely used in previous multicenter radiomics studies for radiomics feature measurement of CT and MRI images [14, 26, 28]. Then, z-scores were calculated to standardize the extracted radiomic features for all three CECT phases. Further elaboration on the role of the combat compensation method and its significance in terms of data normalization in provided in Supplementary Appendix 3.

### Radiomic feature extraction

**Fig. 2:**
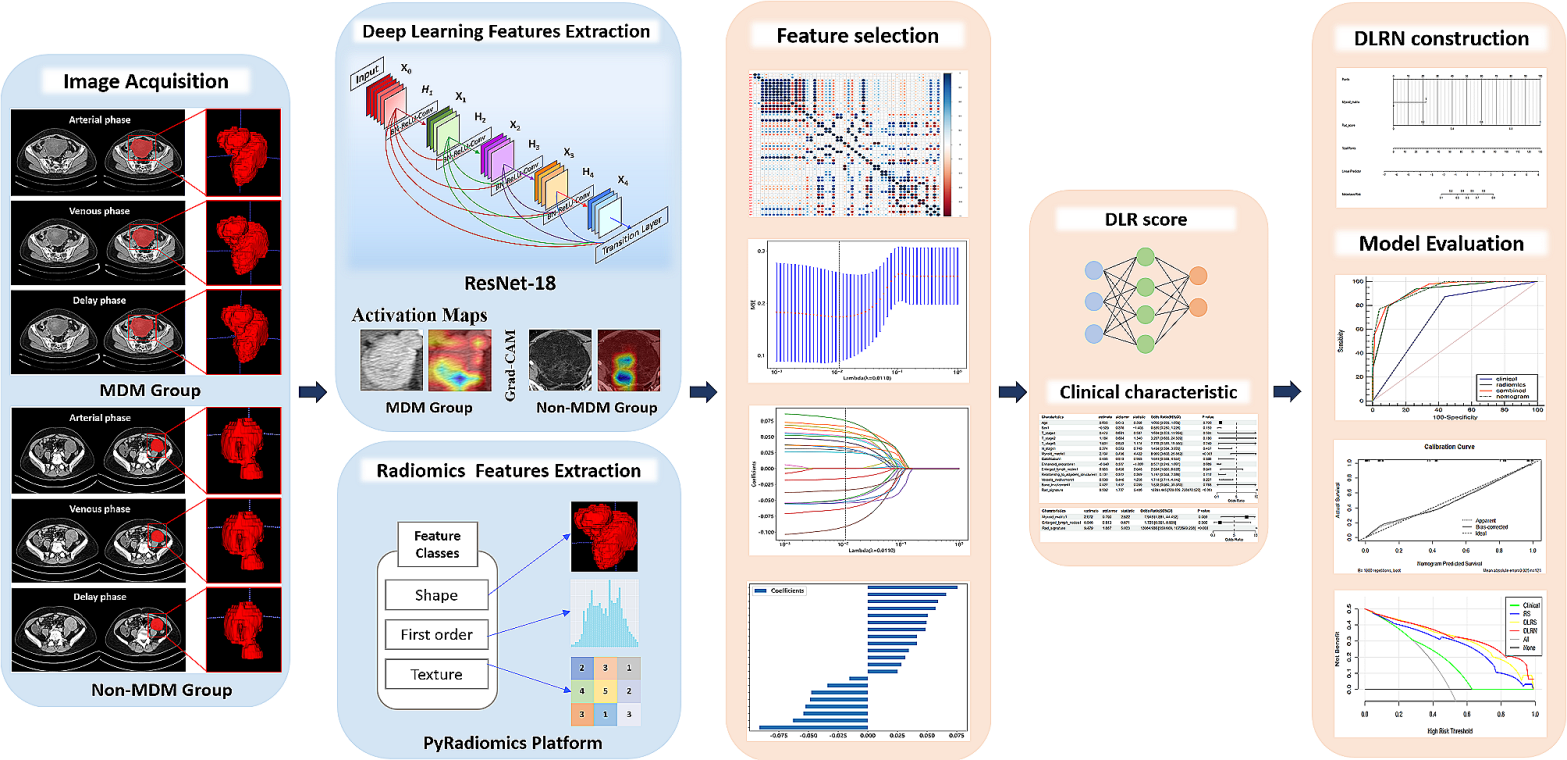
Schematic of the deep learning radiomics analysis

A detailed schematic of the radiomic analysis is presented in Fig. 2. A total of 5,502 hand-crafted radiomic features were extracted from the ROIs of the three-phase CECT images using an open-source Python package (Pyradiomics). For deep learning analytics, a deep learning model with the deep CNN ResNet-18 architecture was pre-trained using the ImageNet dataset based on the PyTorch 1.4.0 framework [29]. In the ResNet-18 model, the output of the penultimate layer of the trained CNN was used to define the deep learning features; after eliminating the null features, 1,536 deep learning features were ultimately extracted from the ROIs of the three-phase CECT images. The details on hand-crafted radiomics and deep learning features extraction are provided in Supplementary Appendix 4.

### Feature selection and construction of the radiomics signature (RS) and the deep learning radiomics signature (DLRS)

Dimensionality reduction of the hand-crafted radiomics features was performed based on the ICCs exceeding a certain threshold (ICCs > 0.8), the minimum redundancy maximum relevance (MRMR) algorithm, and least absolute shrinkage and selection operator (LASSO) logistic regression to select the optimal features, and a RS model was constructed based on the selected features. Furthermore, the deep learning features were screened using the MRMR algorithm, LASSO logistic regression. Finally, a DLRS model was developed by selecting the optimal features from the hand-crafted radiomics and DL features. The DLR-score was calculated for each patient using a linear combination of the selected features weighted by their respective LASSO coefficients.

### Clinical model construction

The clinical model was constructed based on independent clinicoradiological factors predicting MDM identified through univariate and multivariate logistic regression analyses (tumor size > 10 cm, as shown in Table 2). Additionally, the immunohistochemistry findings of Ki67 were obtained from preoperative sarcoma biopsies.

**Table 2:** Univariate and multivariate logistic regression analysis of clinical and CECT semantic features of patients

Variable	Univariate Logistic Analysis		Multivariate Logistic Analysis	
	OR (95% CI)	*P* value	OR (95% CI)	*P* value
DLR-score	13.445 [7.879, 73.527]	< 0.001	13.936 [5.669, 107.238]	< 0.001
Age	1.005 [0.979, 1.033]	0.72	-	-
Sex	0.589 [0.280, 1.225]	0.159	-	-
*Ki-67* index	1.008 [0.990, 1.026]	0.396	-	-
Tumor size > 10 cm	8.969 [3.602, 25.882]	< 0.001	7.943 [1.881, 44.452]	0.009
Clinical N stage	1.454 [0.534, 3.920]	0.457	-	-
Cystic spaces or necrosis	1.604 [0.322, 11.954]	0.591	-	-
Degree of enhancement	1.641 [0.593, 4.551]	0.335	-	-
Enhancement pattern	2.594 [1.050, 6.622]	0.041	1.725 [0.351, 8.909]	0.502
Tumor contours	1.714 [0.711, 4.145]	0.227	-	-
Adjacent organ involvement	1.147 [0.553, 2.386]	0.712	-	-

Abbreviations: DLR, deep learning radiomics; CECT, contrast-enhanced computed tomography; OR, odds ratio; CI, confidence interval

### DLRN construction and performance assessment of the four models

The DLRN model was constructed by combining the independent clinico-radiological predictors with the calculated DLR-score via univariate and multivariate logistic regression analyses. In addition, a clinical model was generated based on the independent clinical-semantic variables described above. The area under the curve (AUC) values were calculated to evaluate the performance of the DLRN, DLRS, RS, and clinical models. The corresponding sensitivity, specificity, accuracy, positive predictive value, and negative predictive value were calculated for each of the three models. Pairwise comparisons of the AUC values of the predictive models were performed using the DeLong test in MedCalc software. Calibration curves were plotted via bootstrapping with 1,000 resamples to evaluate the calibration of the models, and the Hosmer-Lemeshow test was conducted to assess the goodness-of-fit. The clinical usefulness of each model was evaluated via decision curve analysis (DCA) by quantifying the net benefit at various threshold probabilities.

### Follow-up assessments and survival analysis

Distant-metastasis-free survival (DMFS) was defined as the time from surgery to the first appearance of distant metastasis [15]. There is no plateau of distant metastases following surgical resection of RPS, and they may still occur 5–10 years following surgery, requiring long-term close follow-up. Imaging plays a critical role in monitoring disease progression; distant metastases detected on imaging often precede clinical symptoms by months or years. As recommended by the Chinese Society of Clinical Oncology (CSCO) and National Comprehensive Cancer Network (NCCN) guidelines, the follow-up protocol in this study involved abdominopelvic plain + enhanced CT or MRI, along with chest CT every 3 months for the initial year post-surgery. After this period, the imaging frequency can be reduced to every 6 months for 2–5 years, and annually thereafter, depending on the condition of the patient and the availability of resources. Subsequent histopathologic confirmation based on core needle biopsy or surgical specimen tissue ensures the accuracy of the imaging assessment results. The follow-up deadline was May 31, 2023.

Furthermore, an analysis of DMFS times was performed using Kaplan-Meier survival curves, and survival outcomes were analyzed using the log-rank test to compare the DMFS probability of patients in different metastatic risk groups. The DLRN model was included in the DMFS stratification assessment.

## Results

### Baseline information

A total of 179 patients with surgically treated and histologically confirmed RLS were enrolled in the study. MDM occurred in 63 patients, 18 and 23 of whom developed lung and liver metastases, respectively. The median duration of the follow-up period was 19 (13.7–34) months in the training cohort and 15 (12–28) months in the external validation cohort. Comprehensive information regarding the clinical characteristics and semantic features of the tumors based on the CECT images are summarized in Table 1. There were no statistically significant differences between the MDM and non-MDM groups for most variables, including age, sex, and the Ki-67 index, in either the training or validation cohorts; however, significant intergroup differences were observed in the training cohort in terms of tumor size > 10 cm (*P* < 0.001).

### Feature selection and construction of the RS and the DLRS

**Fig. 3:**
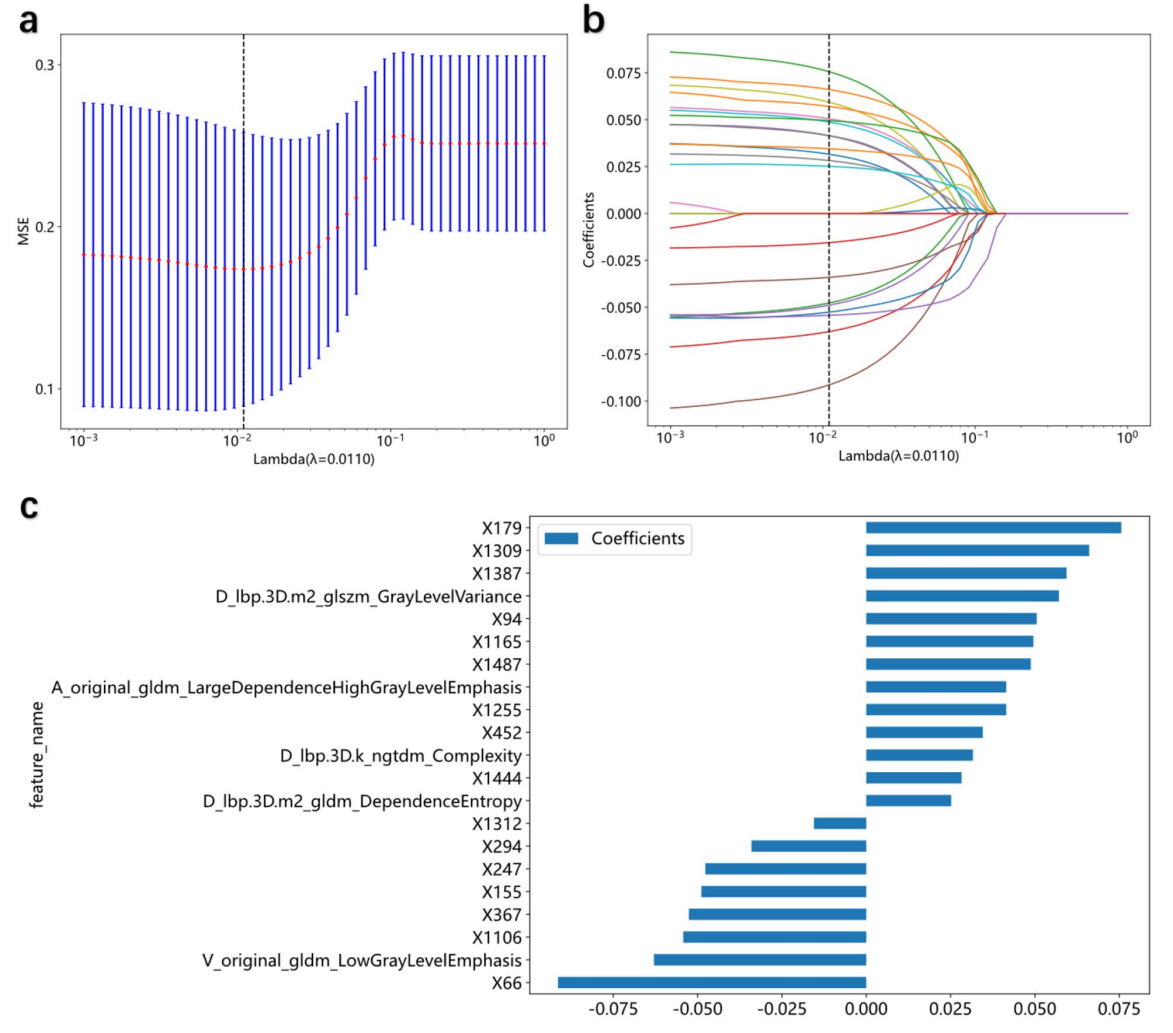
Feature selection for the development of the DLRS using the LASSO regression model with a vertical line generated at the log (λ) value by using ten-fold cross-validation (**a**, **b**); The five radiomics features and 16 deep learning features and their corresponding coefficients (**c**); DLRS, deep learning radiomics signature; LASSO, least absolute shrinkage and selection operator

Overall, 5,329 hand-crafted features from the three-phase CECT images exhibited high reproducibility (ICC > 0.8); in the screening using the MRMR algorithm and LASSO logistic regression (Figures S3a-b), 24 most valuable hand-crafted features (Figures S3c) were selected to develop the RS. For developing the DLRS, 5,329 hand-crafted features were combined with 1,536 deep learning features for inclusion in the subsequent analyses; by using the MRMR algorithm, 25 radiomics/DL features were selected and entered into the LASSO logistic regression model (Fig. 3a-b); finally, 16 deep learning features and five hand-crafted features (Fig. 3c) were combined to construct the DLRS. Subsequently, a DLR-score was calculated for each patient based on a linear combination of the selected features weighted by their respective LASSO coefficients. The formula used to calculate the DLR-score was as follows:

*DLR-score* = 0.39669421487603296 + 0.031597 × *D_lbp-3D-k_ngtMDM_Complexity*

+ 0.066068 × *X1309* + 0.075600 × *X179–0*.015528 × *X1312* + 0.041467 × *X1255*.

+ 0.041481 × *A_original_glMDM_LargeDependenceHighGrayLevelEmphasis*.

+ 0.059346 × *X1387* + 0.048740 × *X1487–0*.091377 × *X66* + 0.050499 × *X94*

+ 0.057080 × *D_lbp-3D-m2_glszm_GrayLevelVariance* − 0.047711 × X247.

− 0.062957× *V_original_glMDM_LowGrayLevelEmphasis*.

− 0.048884 × *X155–0*.034018 × *X294* + 0.028234 × *X1444*.

+ 0.025147 × *D_lbp-3D-m2_glMDM_DependenceEntropy*.

− 0.052596 × *X367* + 0.034525 × *X452* + 0.049526 × *X1165–0*.054271 × *X1106*.

Moreover, the meaning of each variable in the formula is described in the Supplementary Appendix 5–6.

### Development of the DLRN and performance assessment of the four models

**Fig. 4:**
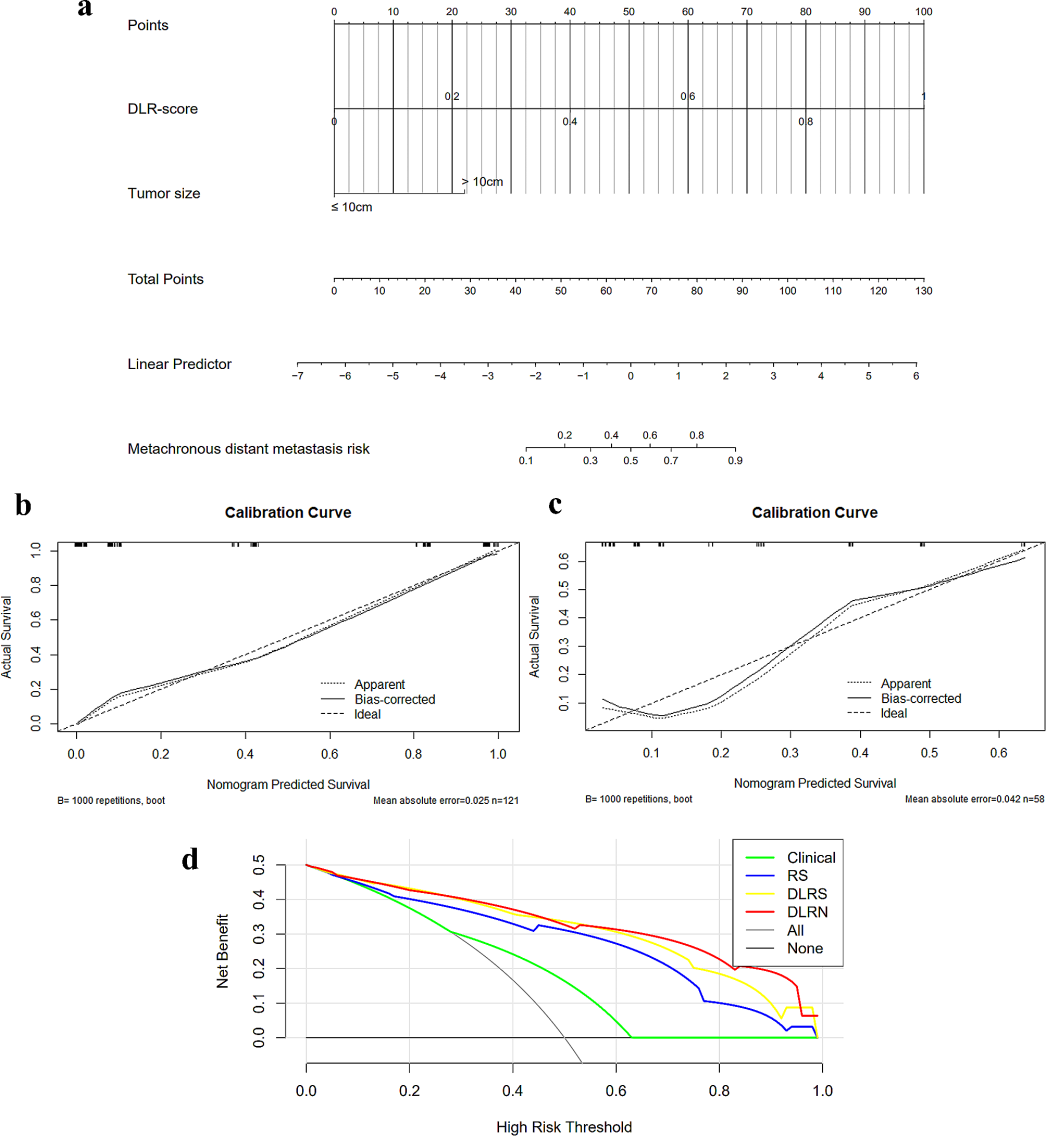
DLRN construction and performance evaluation. (**a**) is a nomogram for individual prediction of MDM risk combined with the DLRS and independent clinico-radiological features; (**b**) and (**c**) are the calibration curves of the DLRN in the training and external validation cohort, respectively; (**d**) are the decision curves of the DLRN, DLRS, RS, and clinical models of the external validation cohort. DLRN, deep learning radiomics nomogram; DLRS, deep learning radiomics signature; RS, radiomics signature; MDM, metachronous distant metastasis

The univariate and multivariate logistic regression analyses revealed that tumor size > 10 cm and the DLR-score were independent predictors of MDM (Table 2). These two variables were incorporated into the DLRN in the training set (Fig. 4a). The AUCs of the training and external validation sets of the DLRN (0.939 and 0.822, respectively) were higher than those of the DLRS (0.937 and 0.786, respectively), RS (0.917 and 0.733, respectively), and clinical models (0.718 and 0.511, respectively) (Table 3). According to the DeLong test, the DLRN, DLRS and RS models all performed significantly better than the clinical model in the training set (both *P* < 0.001) and external validation set (*P* = 0.003, *P* = 0.004, and *P* = 0.015, respectively); however, there was no significant difference in performance between the DLRN and DLRS models (both *P* > 0.05) (Table 3). Furthermore, the DLRN and DLRS models were comparable in terms of their predictive accuracy, specificity, and negative predictive value, and both outperformed the RS and clinical models. As shown in Fig. 4b and c, the calibration curves of the DLRN model indicated good consistency between the predicted and actual probabilities of MDM in both the training and external validation sets. Additionally, the DCA graphically revealed that employing the DLRN model to predict the probability of MDM conferred a better overall net benefit compared with that of the DLRS, RS, and clinical models over the relevant threshold range, indicating that the DLRN exhibited good clinical performance (Fig. 4d).

**Table 3:** Model performances in the training and external validation cohorts

Model	Training Cohort					External Validation Cohort				
	SPE	NPV	ACC	AUC	95% CI	SPE	NPV	ACC	AUC	95% CI
DLRN	0.959	0.868	0.884	0.939	0.905–0.974	0.814	0.921	0.810	0.822	0.692–0.953
DLRS	0.918	0.870	0.868	0.937	0.899–0.975	0.905	0.864	0.810	0.786	0.649–0.923
RS	0.904	0.864	0.860	0.917	0.870–0.964	0.780	0.889	0.741	0.733	0.573–0.892
Clinical model	0.875	0.872	0.686	0.718	0.644–0.793	0.674	0.750	0.500	0.511	0.359–0.662
**DeLong test**	Standard Error		95% CI		*P* value	Standard Error		95% CI		*P* value
DLRN vs. DLRS	0.009		-0.017–0.022		0.816	0.038		-0.037–0.083		0.465
DLRN vs. RS	0.109		-0.160–0.267		0.624	0.031		-0.024–0.096		0.233
DLRN vs. Clinical	0.037		0.148–0.294		< 0.001	0.092		0.094–0.456		0.003
DLRS vs. Clinical	0.042		0.137–0.301		< 0.001	0.109		0.098–0.525		0.004
DLRS vs. RS	0.114		-0.133–0.313		0.429	0.032		-0.042–0.083		0.523
RS vs. Clinical	0.045		0.110–0.287		< 0.001	0.091		0.043–0.401		0.015

Note: The AUCs among models were compared using the DeLong test

Abbreviations: DLRN, deep learning radiomic nomogram; DLRS, deep learning radiomics signature; RS, radiomics signature; SPE, specificity; NPV negative predictive value; ACC, accuracy; AUC, area under the receiver operating characteristic curve; CI, confidence interval

### Individualized prognostic evaluation

**Fig. 5:**
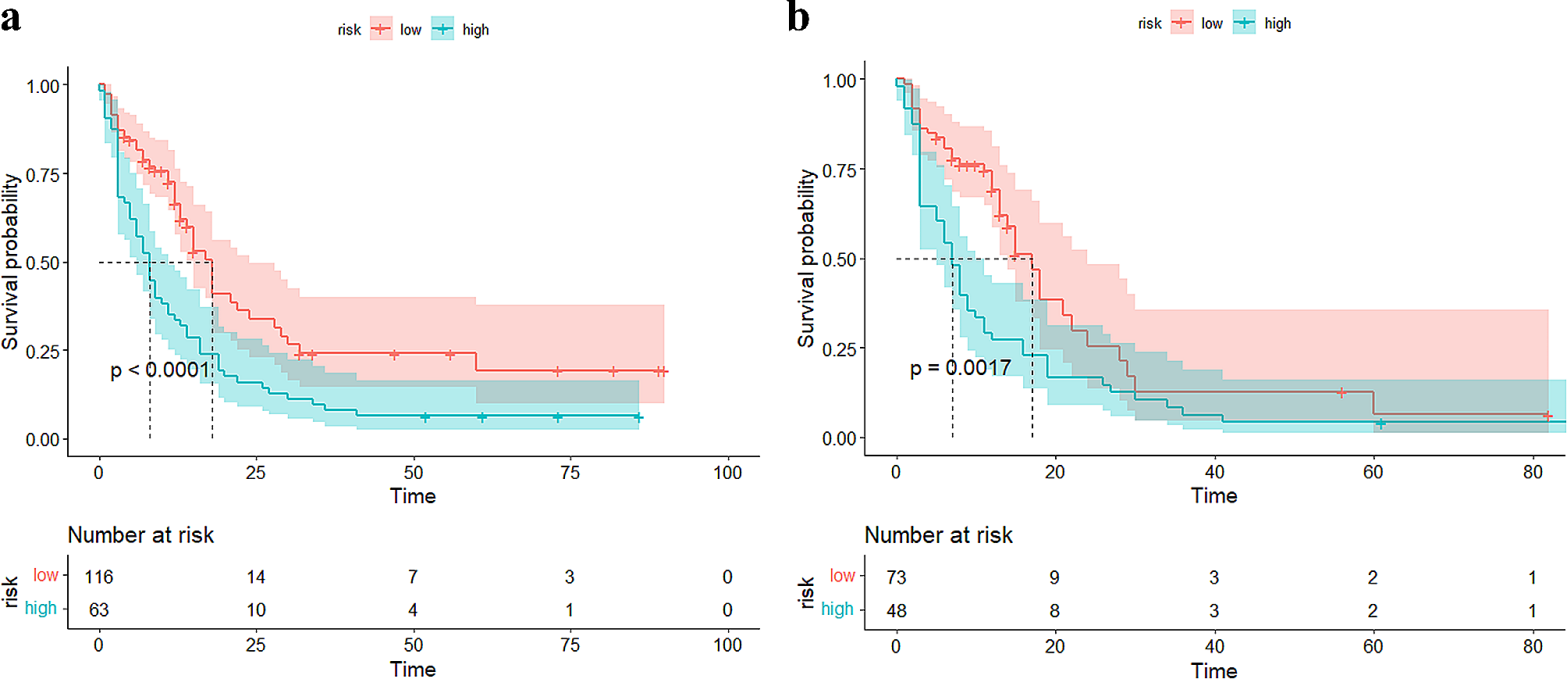
Kaplan-Meier survival analysis curves of distant metastasis-free survival between the groups with low and high DLRN scores in the training (**a**) and external validation cohorts (**b**). DLRN, Deep learning radiomics nomogram

DMFS outcomes were evaluated for all patients using Kaplan-Meier survival curves, which indicated that the DLRN model could be used for risk stratification in both the training and external validation cohorts (Fig. 5a and b), with higher DLRN scores being significantly associated with poorer DMFS (log-rank tests, *P* < 0.001 and *P* = 0.002, respectively).

## Discussion

The aggressive and invasive nature of RLS, combined with the frequency of MDM occurrence, results in a dismal prognosis for patients [1, 5, 13]. In the era of precision medicine, the ability to accurately predict the likelihood of MDM is crucial for facilitating optimal therapeutic decision-making. However, there are currently no reliable tools for predicting such outcomes preoperatively. In this bicentric study, we sought to address this unmet need by developing and validating radiomics methods to extract mineable features from preoperative CECT images. The performances of both the DLRN, DLRS, and RS models were significantly greater than that of the clinical model in predicting the occurrence of MDM, indicating that such radiomics-based approaches are likely to improve upon the current methods for the diagnosis and management of RLS. This validated, noninvasive radiomics model can be utilized by radiologists and surgeons to enhance the accuracy of preoperative MDM predictions and facilitate the design of individualized treatment plans.

Previous studies have reported that age, histological grading, pathological subtype, multifocality, and R0 resection are the main prognostic factors affecting DMFS following surgical resection in patients with RLS [14, 30, 31]. However, in the present study, the clinical model constructed based on the semantic imaging features identified as independent predictors of MDM in the univariate and multivariate analyses (tumor size > 10 cm) had an AUC of 0.511. This value is considerably inferior to that of the radiomics model (0.733–0.786), indicating the limited value of assessing the prognosis of RLS solely based on visual CECT features. Semantic features, such as lesion density, morphology, or size, which can be visually interpreted by radiologists, are predominantly based on empirical judgments. This approach introduces a significant degree of subjectivity in image interpretation and severely limits the consistency and diagnostic efficacy of the model.

Radiomics can overcome the limitations of semantic imaging features through image standardization processing, optimization of feature extraction algorithms, establishment of multi-modal models, enhancement of data sets, and introduction of domain knowledge, which could improve the diagnostic efficiency and consistency of the model. Moreover, the radiomics technique is characterized by automatic representative data acquisition, eliminating the need for clinical index collection, semantic feature interpretation, and manual annotation. Therefore, radiomics is readily accepted in clinical workflow. Radiomics can noninvasively capture risk-related intra- and inter-tumoral heterogeneity at the voxel level, providing a more objective and thorough means of characterizing sarcomas in a clinical setting [18, 32].

Radiological imaging plays a key role in ascertaining the likelihood of radical surgical resection, given its ability to determine the precise anatomical relationship between a mass and key retroperitoneal organs and vascular structures. The NCCN guidelines recommend the use of CECT for monitoring metastasis in patients with RLS, as its occurrence can potentially impact a patient’s prognosis and treatment options [13]. Additionally, Cui et al. [33] reported that the performance of a multiphase CT-based model was superior to that of models that relied on single-phase imaging in the assessment of International Society of Urologic Pathologists (ISUP) grading for clear cell renal cell carcinoma. Ni et al. [34] also reported the benefits of using a combined model to distinguish between sclerosing pneumocytomas and solid malignant pulmonary nodules. These findings led to the construction of the predictive radiomics model described in the present study, which was based on three-phase CECT features and exhibited broad representation and robust performance.

Given the potential of radiomics and the limitations associated with the use of conventional hand-crafted features, there has been a surge in studies investigating methods for the identification and adaptive and automated extraction of radiomic features in a data-driven manner using deep learning methods, particularly those involving CNNs [20]. In contrast to methods that involve the selection of hand-crafted features, the deep learning approach does not necessitate contouring, which not only reduces contour variability across manual segmentations, but also enhances efficiency. In addition, deep learning provides more detailed information, including that related to specific tasks in the hidden layer of a neural network, without requiring predefined features [22]. Thus, the deep learning approach partially compensates for the limitations associated with methods based on hand-crafted radiomics features. Previous studies have demonstrated that an integrated model combining deep learning and hand-crafted radiomics features could exhibit superior performance in predicting tumor prognosis compared with that of models based on either approach alone [21]. In this study, we focused on effectiveness and simplicity; we utilized the MRMR and LASSO algorithms for feature selection in combination with a machine learning classifier (KNN) to develop an integrated DLRS model for predicting the risk of MDM after surgical resection to treat RLS. The KNN algorithm is one of the simplest and most well-established data mining classification techniques, reducing the risk of misclassification by maximizing the space between the plane and various types of data points. Furthermore, the DLRS model yielded better auxiliary predictions than the RS and clinical models, as indicated by its higher AUC and accuracy. In addition, the DLRS model included 16 deep learning features, indicating that the CNNs may have captured quantitative information reflecting the risk of MDM in patients with RLS. The lack of interpretation of deep learning feature is a major obstacle to the practical application of deep learning models in clinical practice. A common approach to improving the interpretation of deep learning feature is to generate visual feature CNN Activation Maps and explore the decision-making implications of the attention regions [35–37]. As shown in Figure S4, the Activation Maps highlighted certain parts within the tumors with high predictive value in determining the MDM status. Typically, regions with high heat indicate areas of tumors that are abnormally active [35]. These regions may be characterized by features such as tumor size, shape, density, blood flow, and others. Broadly speaking, the Activation Maps of MDM tumors appear busier in comparison to those of non-MDM tumors, which look sparser. Such visual pattern may make the process less of a “black box” and increase the interpretability of the machine diagnosis. We hypothesized that the areas highlighted in Activation Maps would exhibit greater correlations with the likelihood of MDM occurrence.

The significantly relevant clinico-radiological characteristic (tumor size > 10 cm) was subsequently combined with the DLRS model to establish a highly accurate DLRN, which outperformed both the DLRS and clinical models in predicting the occurrence of MDM, as evidenced by its excellent clinical utility. Another intriguing finding was the satisfactory risk stratification performance of the DLRN for determining DMFS outcomes in both the training and external validation cohorts, highlighting the fact that patients with MDM experience a poorer prognosis.

Surgical resection remains the cornerstone of treatment for localized RLS [13]; however, achieving an R0 resection with microscopically negative margins remains challenging. Owing to its rarity and complexity, centralized treatment of RPS in dedicated sarcoma centers entails a coordinated approach among multiple healthcare professionals. This approach can improve outcomes by ensuring that patients receive the most advanced and comprehensive treatment options. A previous nationwide study reported that the prognosis of patients with RLS may be impacted by the case volume and expertise of the treating facility [38]. Bonvalot et al. [39] reported that patients treated surgically at specialty sarcoma centers exhibited significantly better OS than those treated at non-centers, with 2-year OS of 87% vs. 70%, respectively (*P* < 0.001); notably, the multivariate analysis identified treatment at a specialized center as an independent predictor of OS, with a twofold lower odds ratio of death. Gutierrez et al. [40] also reported a higher OS for patients with retroperitoneal sarcoma treated in a dedicated sarcoma center (39 vs. 31 months, *P* = 0.011). Despite advancements in surgical techniques, the prevalence of MDM remains high, highlighting the desperate need for additional therapeutic strategies, such as multi-modal therapy for RLS. Doxorubicin and ifosfamide are commonly used in first-line postoperative chemotherapeutic regimens [13]; however, no standardized treatment regimen involving currently available agents has been established to date. Although there is no available evidence to confirm the benefit of neoadjuvant chemotherapy, it may be an option for patients with a high risk of developing MDM, following discussions with a multidisciplinary team of physicians. Notably, a phase III randomized controlled trial (NCT04031677) is currently underway to evaluate the clinical benefits of neoadjuvant chemotherapy preceding surgery, the results of which are expected to be reported in 2027 [1]. Radiotherapy offers a potential means of eliminating residual micro-metastases, although its use remains controversial. For example, the results of a phase III clinical trial (EORTC62092) revealed that preoperative radiotherapy did not prolong disease-free survival, and most guidelines do not currently recommend its use as a regular treatment option for various sarcomas [41]. Aiba et al. [42] reported that combined radiotherapy and hyperthermic chemotherapy may also be an effective option for salvage treatment of residual lesions. Additionally, promising outcomes have been observed in patients with high-risk RPS treated with targeted therapies such as pazopanib and olaratumab, as well as with novel immunotherapies such as nivolumab and pembrolizumab [43]. Studies investigating the role of the tumor microenvironment, tumor-infiltrating lymphocytes, and tumor-mutated genes have provided insights into the interactions between sarcomas and immunotherapeutic agents and identified potential targets for immunotherapy that should be further assessed in future clinical trials [44, 45]. The landscape of RLS treatment is moving toward personalized therapy, early intervention, the expansion of drug options, and better survival outcomes. Thus, the sarcoma team can noninvasively and accurately identify people at high risk for MDM by using the DLRN proposed in this study to guide personalized adjuvant treatment regimens and improve clinical outcomes, and potentially achieve the ultimate goal of precision medicine.

This study has some limitations that merit consideration. First, inherent bias cannot be eliminated owing to the retrospective design. Prospective validation of the deep learning radiomics model is necessary to confirm its generalizability and clinical utility. Second, the sample size may have been relatively small because of the rarity of RLS; considering the small sample validation cohort, the real differentiation efficiency of clinical variables would be easily interfered with inevitable inter-cohort bias causing the unpredicted reduction of diagnostic efficiency. Finally, the biological significance of the selected radiomics features remains to be elucidated, and future research integrating imaging modalities with molecular, epigenetic, or transcriptional data may provide greater insight into micro-information and the relationships with other variables.

## Conclusions

The novel DLRN constructed in this study, which combined deep learning radiomics features and semantic tumor features based on CECT imaging, exhibited promising performance in the preoperative prediction of the risk of MDM following curative resection in patients with RLS. This nomogram can provide valuable information that would allow dedicated sarcoma teams to better tailor personalized treatment plans and improve clinical outcomes. Further investigations from multiple centers are warranted to validate this model before its future clinical application.

### Electronic supplementary material

Below is the link to the electronic supplementary material.

Supplementary Material 1

Supplementary Material 2

Supplementary Material 3

Supplementary Material 4

## Data Availability

Due to the privacy of patients, the raw data cannot be available for public access but can be obtained from Daorong Wang (wdaorong666@sina.com) upon reasonable request.

## Abbreviations

AJCC: American Joint Committee on Cancer
CECT: Contrast-enhanced computed tomography
CNN: Convolutional neural network
DCA: Decision curve analysis
DLRN: Deep learning radiomics nomogram
DLRS: Deep learning radiomic signature
DMFS: Distant metastasis-free survival
ICC: Intraclass correlation coefficient
ISUP: International Society of Urologic Pathologists
KNN: k-nearest neighbors
LASSO: Least absolute shrinkage and selection operator
MDM: Metachronous distant metastasis
MRMR: Minimum redundancy maximum relevance
NCCN: National Comprehensive Cancer Network
RLS: Retroperitoneal leiomyosarcoma
ROI: Region of interest
RPS: Retroperitoneal sarcomas
TNM: Tumor, node, metastasis
