# Supplementary material for: Deep learning radiomics-based prediction model of metachronous distant metastasis following curative resection for retroperitoneal leiomyosarcoma: a bicentric study
Source: Cancer Imaging. 2024 Apr 16;24:52. doi: 10.1186/s40644-024-00697-5 (PMC11020328; doi:10.1186/s40644-024-00697-5)
Supplement: Supplementary file 1 — Supplementary Material 1 [file 40644_2024_697_MOESM1_ESM.doc]

[Date of submission: October 10th, 2023]

Prof. Rodney Hicks & Prof. Annick Van den Abbeele

Editors-in-Chief

*Cancer Imaging*

Dear Editors:

I wish to submit an original research article for publication in *Cancer Imaging*, titled “Deep Learning Radiomics-Based Prediction Model of Metachronous Distant Metastasis Following Curative Resection for Retroperitoneal Leiomyosarcoma: A Bicentric Study.” The paper was coauthored by Zhen Tian, Yifan Cheng, Shuai Zhao, Ruiqi Li, Jiajie Zhou, Qiannan Sun, and Daorong Wang.

Combining conventional radiomic models with deep learning features can result in superior performance in predicting the prognosis of patients with tumors; however, this approach has never been evaluated for the prediction of metachronous distant metastasis (MDM) among patients with retroperitoneal leiomyosarcoma (RLS). Thus, the purpose of this study was to develop and validate a preoperative computed tomography (CT)-based deep learning radiomics model for predicting the occurrence of MDM in patients with RLS undergoing complete surgical resection.

This retrospective, bicentric study included 179 patients who had undergone surgery for the treatment of RLS, the diagnosis of which was histologically confirmed post-surgery. Semantic segmentation features derived from a convolutional neural network deep learning model as well as hand-crafted radiomic features were extracted from preoperative three-phase contrast-enhanced CT images to quantify the sarcoma phenotypes. A deep learning radiomic signature (DLRS) model that incorporated radiomic and deep learning features was developed to predict the risk of MDM. Additionally, a deep learning radiomics nomogram (DLRN) was established to evaluate the incremental prognostic significance of the DLRS in combination with clinico-radiological predictors. Both the integrated DLRN and DLRS models exhibited superior predictive performance compared with that of the clinical model. The decision curve analyses indicated that utilizing the DLRN for risk stratification provided greater net benefits than those achieved using the DLRS and clinical models. Good alignment with the calibration curve indicated that the DLRN also exhibited good performance.

We believe this study is relevant and will be of interest to the readers of your journal, as we have developed and validated a novel CT-based DLRN that exhibited better performance in predicting the risk of MDM following curative resection in patients with RLS compared with that of the clinical model based on conventional semantic tumor features. Surgical resection remains the only potentially curative treatment option for RLS, and MDM is common even in cases of R0 resection, resulting in very poor survival outcomes in this patient population. Thus, the novel model we have developed and validated is expected to provide valuable information to help physicians predict the surgical efficacy and tailor individualized treatment plans in these patients, while also helping to identify patients with a low risk of MDM, thereby minimizing the adverse effects of over-monitoring and over-treatment.

This manuscript has not been published or presented elsewhere in part or in entirety and is not under consideration by another journal. The study design was approved by the appropriate ethics review board. We have read and understood your journal’s policies, and we believe that neither the manuscript nor the study violates any of these. There are no conflicts of interest to declare.

Thank you for your consideration. I look forward to hearing from you.

Sincerely,

[Corresponding author: Daorong Wang]

[Affiliation: Department of General Surgery, Northern Jiangsu People's Hospital]

[Postal address: No. 98, Nantong West Road, Yangzhou City, Jiangsu Province, China]

[Email address: [wdaorong666@sina.com](mailto:wdaorong666@sina.com)]
