# Supplementary material for: Deep learning radiomics-based prediction model of metachronous distant metastasis following curative resection for retroperitoneal leiomyosarcoma: a bicentric study
Source: Cancer Imaging. 2024 Apr 16;24:52. doi: 10.1186/s40644-024-00697-5 (PMC11020328; doi:10.1186/s40644-024-00697-5)
Supplement: Supplementary file 2 — Supplementary Material 2 [file 40644_2024_697_MOESM2_ESM.docx]

**Supplementary Materials:**

**Table S1.** The interobserver agreement of CECT semantic features for retroperitoneal leiomyosarcoma

| CECT semantic feature | Disagreement | Kappa value/ICC | 95% CI |
| --- | --- | --- | --- |
| Tumor size | 10 (5.6) | 0.858 | 0.806-0.908 |
| CECT-reported LN status | 4 (2.2) | 0.937 | 0.902-0.976 |
| Cystic spaces or necrosis | NA | 0.985 | 0.975-0.990 |
| Degree of enhancement | 9 (5.0) | 0.846 | 0.801-0.892 |
| Enhancement pattern | 7 (3.9) | 0.863 | 0.826-0.900 |
| Tumor contours | 3 (1.7) | 0.947 | 0.922-0.972 |
| Adjacent organ involvement | NA | 0.989 | 0.979-0.993 |

Abbreviations: CECT, contrast-enhanced computed tomography; CI, confidence interval. ICC, intraclass correlation coefficient.

**Appendix 1:** Cohen’s Kappa coefficient and intraclass correlation coefficient (ICC).

Generally, the Kappa coefficient/ICC of 0–0.20 indicates a poor agreement; 0.21–0.40 fair agreement; 0.41–0.60 moderate agreement; 0.61–0.80 good agreement, > 0.80 excellent agreement.

**Appendix 2:** CECT scans were conducted using one of the following helical CT scanners: Optima CT 620, Optima CT 670, Revolution CT, or LightSpeed CT750 HD (GE Healthcare); or SOMATOM® Sensation 64, or Definition Flash (Siemens).

The following scanning parameters were employed: tube voltage, 120 kV; tube current, 240–340 mA (50 mA for pediatric patients); matrix, 512×512; pitch, 1.375; section thickness, 5 mm; and observation via soft tissue window. Non-ionic contrast medium (iopromide, Ultravist 370; Bayer) was intravenously administered at a dose of 1.5 mL/kg body weight and a rate of 3 mL/s (1.5–2.0 mL/s in pediatric patients). Arterial (25-30s), venous (60s), and delayed-phase (2-3min) images were collected in each patient.

**Appendix 3:** Segmenting images into Region of Interest (ROI), such as tumors, normal tissues, and other anatomical structures, is an essential step that lays the foundation for subsequent data analysis. In the past 20 years, many popular segmentation algorithms have been applied in medical imaging research, with manual segmentation methods often used as a gold standard or ground truth. Before conducting this research, we referenced published methods for region of interest (ROI) segmentation in some radiomics studies. For example, Nie et al [1], Yang et al [2], and Wang et al [3] all used manual segmentation to delineate ROIs, and their findings revealed that the radiomics features extracted by this method were valuable for prognosis prediction; therefore, we also took a manual segmentation approach. Automatic segmentation, however, remains controversial and its accuracy requires further evaluation, as it often introduces significant variations in segmentation observed between different observers. Manual tracking boundaries also has its own inherent biases and low reproducibility of imaging experts, and is time-consuming. It is still necessary to establish a standardized, automated, and reproducible segmentation algorithm.

**References**

1. Nie, P., J. Zhang, W. Miao, S. Duan, T. Wang, J. Zhang, et al., Incremental value of radiomics-based heterogeneity to the existing risk criteria in predicting recurrence of hepatocellular carcinoma after liver transplantation. Eur Radiol, 2023. 33(9): p. 6608-6618.

2. Yang, G., P. Nie, L. Yan, M. Zhang, Y. Wang, L. Zhao, et al., The radiomics-based tumor heterogeneity adds incremental value to the existing prognostic models for predicting outcome in localized clear cell renal cell carcinoma: a multicenter study. Eur J Nucl Med Mol Imaging, 2022. 49(8): p. 2949-2959.

3. Wang, H., M. Zhang, J. Miao, F. Hou, Y. Chen, Y. Huang, et al., Deep learning signature based on multiphase enhanced CT for bladder cancer recurrence prediction: a multi-center study. EClinicalMedicine, 2023. 66: p. 102352.

**Appendix 4:** The main purpose of using ComBat compensation method is to eliminate batch effects caused by technical variations or differences in scanning devices and parameters between different image batches. These batch effects can lead to differences in feature representation between different batches of image data, which can affect the accuracy and reliability of the results. ComBat method corrects the data to make the image data from different batches more comparable and improves the interpretability of research results by preserving the biological differences between samples.

The main advantages of ComBat compensation method include:

1. Correcting batch effects: ComBat method can effectively correct the batch effects caused by technical variations or instrument differences between different batches, making the data more comparable.

2. Preserving biological differences: ComBat method corrects batch effects while trying to preserve biological differences between samples. This means that important biological information is still retained during the correction process, which helps to more accurately interpret research results.

3. Flexibility and universality: ComBat method is a general batch effect correction method that can be applied to various types of image genomics data, including gene expression data, protein expression data, metabolite data, etc. It has high flexibility and is suitable for various different research designs and data types.

In summary, ComBat compensation method plays an important role in eliminating batch effects, enhancing data comparability, and improving the interpretability of research results in image genomics. It is a commonly used and effective data analysis tool.

**Appendix 5:** The details on hand-crafted radiomics and deep learning features extraction.

For RS model constructed, the hand-crafted features were extracted from the ROIs of three-phase CECT images using an open-source Python package (Pyradiomics), including (1) intensity statistic features, including features that quantitatively delineate the distribution of voxel intensities within the ROIs through commonly used and basic metrics; (2) shape features, including 3-D features, which reflect the shape and size of the ROIs; (3) texture features, which are composed of features calculated by gray level co-occurrence matrix (GLCM), gray level run length matrix (GLRLM), gray level size zone matrix (GLSZM), gray level dependence matrix (GLDM), and neighboring gray tone difference matrix (NGTDM) quantifying the heterogeneity differences of ROIs; and (4) filter and wavelet features, which include the intensity and texture features derived from filter transformation and wavelet transformation of the original images, obtained by applying filters such exponential, logarithm, square, square root, gradient, lbp_2D, lbp_3D_k and wavelet (wavelet-LHL, wavelet-LHH, wavelet-HLL, wavelet-LLH, wavelet-HLH, wavelet-HHH, wavelet-HHL and wavelet-LLL). The instructions of theradiomics features are shown in Pyradiomics document (Version 3.0) on https://pyradiomics.readthedocs.io.

For deep learning analysis, the CECT images were set to soft tissue window (W:350, L:35). The largest cross section was derived from the 3D-ROI, and this ROI was extended outward into a square area. The cropped area was resized to 224×224 pixels as the input channels of ResNet18. The pre-trained ResNet18 model in the Pytorch framework was implemented. DL features were extracted from the largest cross-section of the 3D-ROI, by the pre-trained classification model ResNet18. In the ResNet-18 model, the output of the penultimate layer of the trained CNN was used to define the deep learning features; after eliminating the null features, 1,536 deep learning features were ultimately extracted from the ROIs of the three-phase CECT images.

**Appendix 6:** The meaning of each variable in the formula used to calculate the DLR-score.

(1) The following are hand-crafted radiomics features:

*D_lbp-3D-k_ngtMDM_Complexity*

*A_original_glMDM_LargeDependenceHighGrayLevelEmphasis*

*D_lbp-3D-m2_glszm_GrayLevelVariance* - 0.047711 × X247

*V_original_glMDM_LowGrayLevelEmphasis*

*D_lbp-3D-m2_glMDM_DependenceEntropy*

In which.

1. A / D / V represents the features extracted from arterial / venous / delayed phase CECT images;

2. These five formula coefficients (0.031597, 0.041481, 0.057080, 0.062957, 0.025147) represent the weights of the hand-crafted radiomics features;

3. Radiomics features extracted by Pyradiomics

| Radiomics Feature | Characteristic |
| --- | --- |
| LowGrayLevelEmphasis | This feature is used to measure the distribution and emphasis of pixels at lower gray levels in an image. This means that the feature captures the spatial relationship between the pixels at low gray levels in an image, usually reflecting the texture characteristics of the darker areas of the image. |
| LargeDependenceHighGrayLevelEmphasis | This feature can help analyze the dependencies and emphasis between different regions of grayscale levels in an image. This means that when higher grayscale levels appear, there is a strong correlation between them, possibly representing a specific texture or structural feature. |
| Complexity | Complexity reflects the complexity of texture in an image. Typically, the higher the complexity, the more complex the texture and structure in the image, such as more variations in color, shape, size, etc. |
| GrayLevelVariance | GrayLevelariance measures the variation in gray levels between different pixels in an image. The larger the variance, the more significant the variation in gray levels, indicating a more rich and complex texture in the image, such as more color, shape, size, and other variations. Conversely, a smaller variance indicates a relatively uniform and simple texture in the image. |
| DependenceEntropy | DependenceEntropy measures the uncertainty between pixels in an image. It considers the spatial relationships between pixels and the dependencies between grayscale levels, providing information about image textures. A higher Dependence Entropy value indicates more complex and chaotic textures in the image, while a lower value indicates more uniform and regular textures. |

These features are all measurement indicators used to describe image texture features, and are widely applied in medical image analysis, especially in the areas of cancer diagnosis, recurrence and metastasis prediction, and treatment response assessment. They can provide information about tissue structure, tumor morphology, and internal heterogeneity of tissues, which can help physicians and researchers to detect abnormal regions in images, assess the severity of diseases, extract information about disease states and tissue characteristics, and provide support for clinical decision making.

Notably, combined analysis of these features provides a more comprehensive understanding of the texture features and related information in the image.

(2) The following are deep learning features: *X1309, X179, X1312, X1255, X1387, X1487, X66, X94, X247, X155, X294, X1444, X367, X452, X1165, X1106.*

These deep learning features are output from the penultimate layer of the trained convolutional neural networks. I apologize for the fact that, so far, deep learning features are still not interpretable. The lack of interpretation of deep learning feature is a major obstacle to the practical application of deep learning models in clinical practice. A common approach to improving the interpretation of deep learning feature is to generate visual feature CNN activation maps and explore the decision-making implications of the attention regions [1-3]. As shown in Figure S4, the activation maps highlighted certain parts within the tumors with high predictive value in determining the MDM status. Typically, regions with high heat indicate areas of tumors that are abnormally active [1]. . These regions may be characterized by features such as tumor size, shape, density, blood flow, and others. By analyzing these features, we can gain a better understanding of tumor growth and spread, thus predicting MDM status. Broadly speaking, Activation Maps of MDM tumors appear busier in comparison to Activation Maps of non-MDM tumors, which look sparser. The potential for such visual pattern recognition on Activation Maps by a human expert, to augment the machine learning analysis of the Activation Maps, may make the process less of a “black box” and increase the interpretability of the machine diagnosis. And we will endeavor to explore the interpretability of deep learning features by combining genetic and pathological information in future studies.

**References**

1. Song, H., S. Yang, B. Yu, N. Li, Y. Huang, R. Sun, et al., CT-based deep learning radiomics nomogram for the prediction of pathological grade in bladder cancer: a multicenter study. Cancer Imaging, 2023. 23(1): p. 89.

2. Mazin, A., S.H. Hawkins, O. Stringfield, J. Dhillon, B.J. Manley, D.K. Jeong, et al., Identification of sarcomatoid differentiation in renal cell carcinoma by machine learning on multiparametric MRI. Sci Rep, 2021. 11(1): p. 3785.

3. Kundu, R., P.K. Singh, S. Mirjalili, and R. Sarkar, COVID-19 detection from lung CT-Scans using a fuzzy integral-based CNN ensemble. Comput Biol Med, 2021. 138: p. 104895.


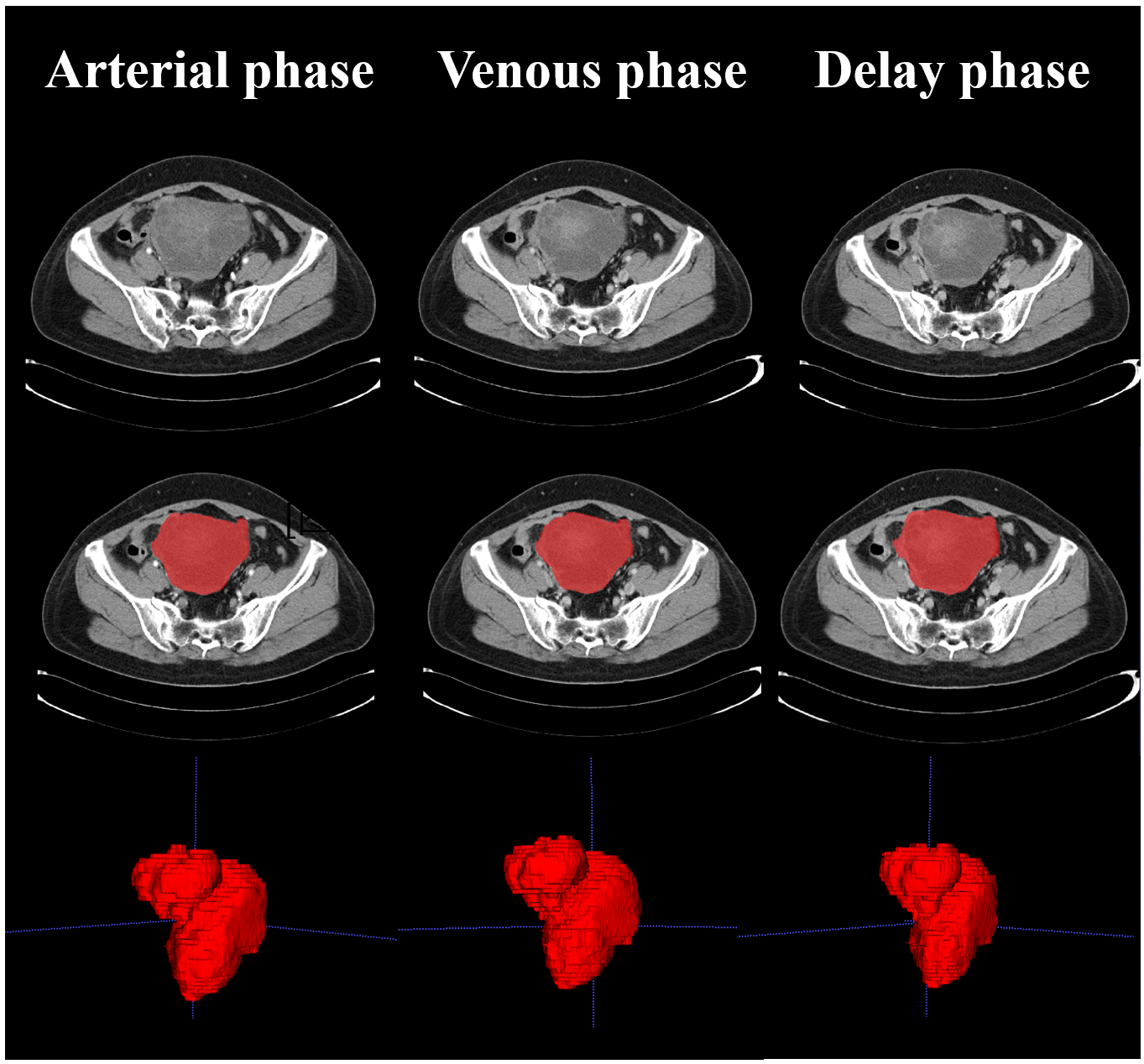


**Figure S1.** Three-dimensional delineation of the retroperitoneal leiomyosarcoma presenting with distant metastasis after surgery was performed manually on the arterial, venous, and delayed phases of CECT images.

**
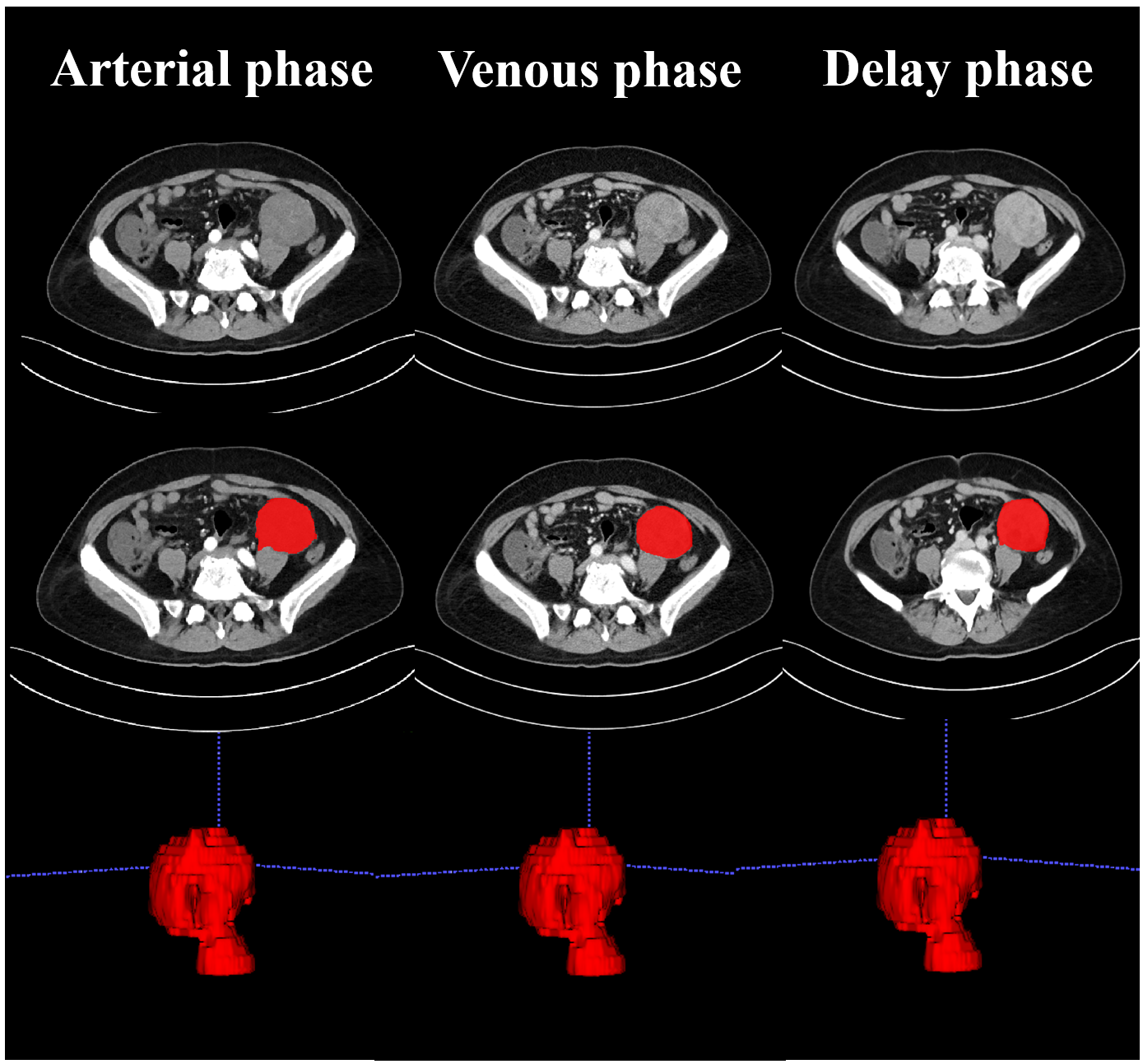
**

**Figure S2.** Three-dimensional delineation of the retroperitoneal leiomyosarcoma without detectable distant metastasis was performed manually on the arterial, venous, and delayed phases of CECT images.


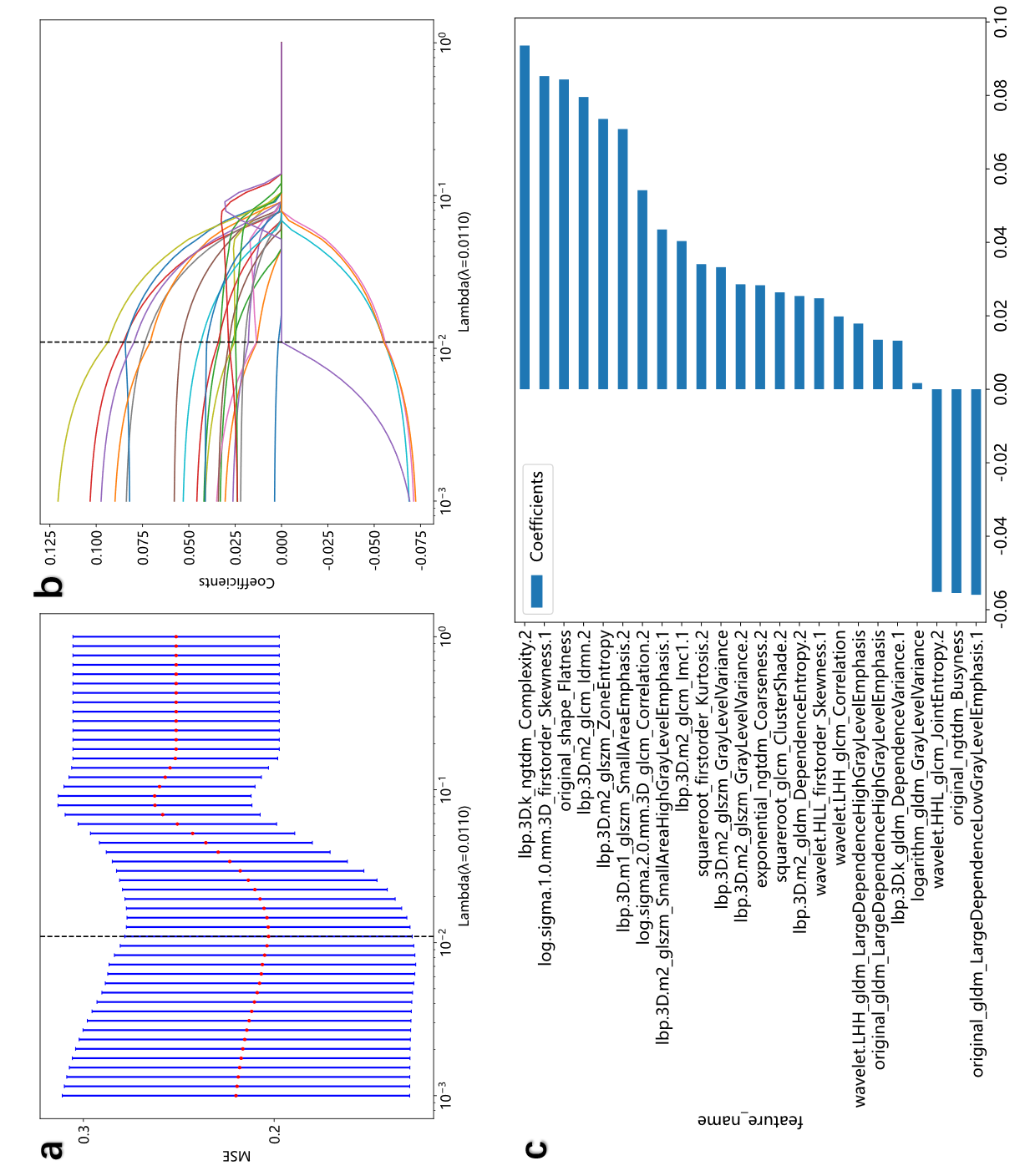


**Figure S3.** Feature selection for the development of the radiomics signature (RS) using the least absolute shrinkage and selection operator regression model with a vertical line generated at the log (λ) value by using ten-fold cross-validation (a, b); The 24 radiomics features and their corresponding coefficients (c).

**
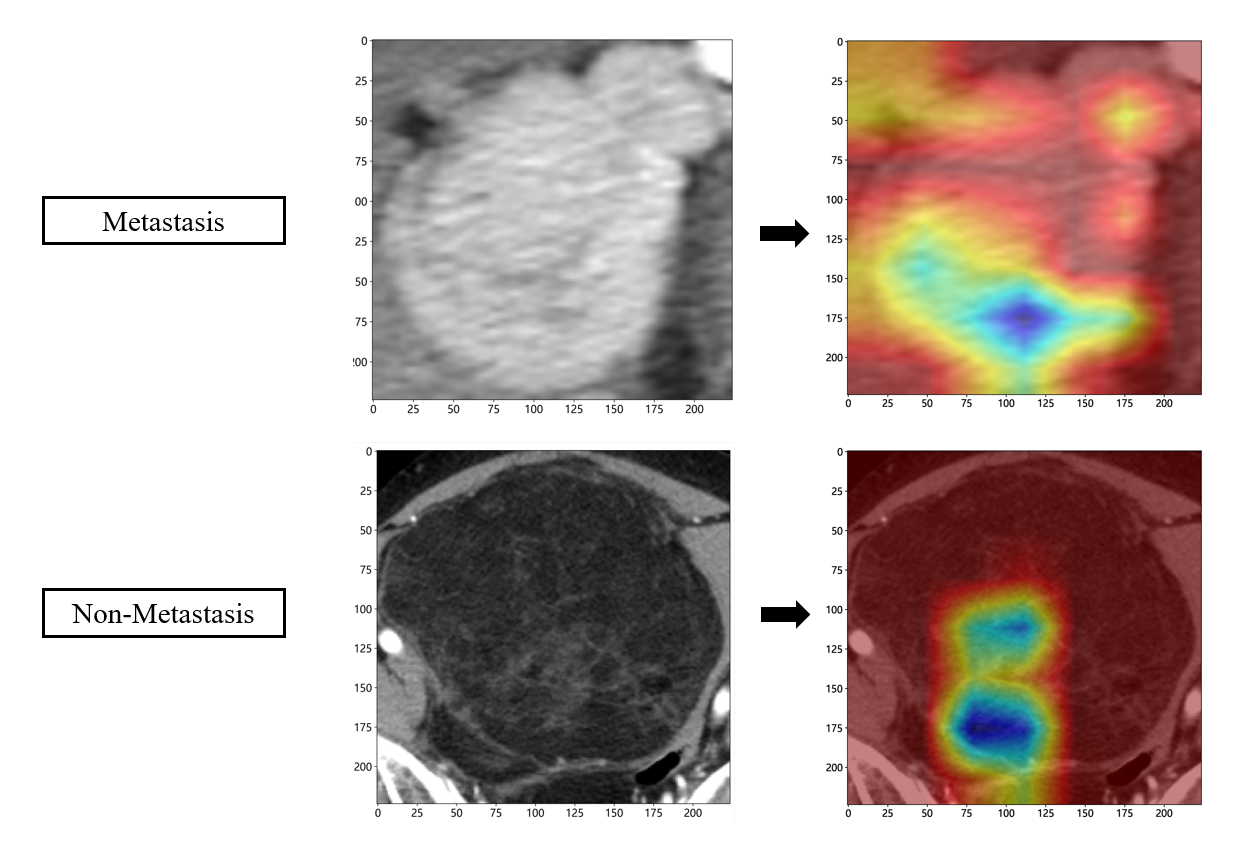
**

**Figure S4** presents the Activation Maps showing important retroperitoneal leiomyosarcoma regions that the deep convolutional neural networks learned as the most predictive of distant metastasis. The highlighted areas represent the regions of high value in the model’s distant metastasis prediction, whereas the suppressed areas are regions with little important predictive value for distant metastasis.
