# Supplementary material for: Deep learning radiomics-based prediction model of metachronous distant metastasis following curative resection for retroperitoneal leiomyosarcoma: a bicentric study
Source: Cancer Imaging. 2024 Apr 16;24:52. doi: 10.1186/s40644-024-00697-5 (PMC11020328; doi:10.1186/s40644-024-00697-5)
Supplement: Supplementary file 3 — Supplementary Material 3 [file 40644_2024_697_MOESM3_ESM.pdf]

CERTIFICATE OF ENGLISH EDITING

This document certifies that the paper listed below has been edited to ensure that the language is clear and free of errors. The logical presentation of ideas and the structure of the paper were also checked during the editing process. The edit was performed by professional editors at Editage, a division of Cactus Communications, in cooperation with Taylor & Francis Group. The intent of the author's message was not altered in any way during the editing process. The quality of the edit has been guaranteed, with the assumption that our suggested changes have been accepted and have not been further altered without the knowledge of our editors.

Title

Deep Learning Radiomics-Based Prediction Model of Metachronous Distant Metastasis Following Curative Resection for Retroperitoneal Leiomyosarcoma: A Bicentric Study

Authors

Zhen Tian, Yifan Cheng, Shuai Zhao, Ruiqi Li, Jiajie Zhou, Qiannan Sun, and Daorong Wang

Order No.

OYLYZ_1_3

EDITINGSERVICES
Supporting Taylor & Francis authors

Signature

Prabh Grewal,
Senior Vice President,
Editage

Date of Issue
February 23, 2024

support@tandfeditingservices.com
