# Supplementary material for: Deep learning radiomics-based prediction model of metachronous distant metastasis following curative resection for retroperitoneal leiomyosarcoma: a bicentric study
Source: Cancer Imaging. 2024 Apr 16;24:52. doi: 10.1186/s40644-024-00697-5 (PMC11020328; doi:10.1186/s40644-024-00697-5)
Supplement: Supplementary file 4 — Supplementary Material 4 [file 40644_2024_697_MOESM4_ESM.docx]

[Date of submission: February 25, 2024]

Prof. Rodney Hicks & Prof. Annick Van den Abbeele

Editors-in-Chief

*Cancer Imaging*

Dear Editors:

On behalf of all contributing authors, we would like to express our sincere appreciation for your letter and the constructive comments provided by the reviewers regarding our article titled " Deep Learning Radiomics-Based Prediction Model of Metachronous Distant Metastasis Following Curative Resection for Retroperitoneal Leiomyosarcoma: A Bicentric Study." (Manuscript No: CAIG-D-23-00558).

The reviewers' comments have proved to be invaluable and have greatly assisted in enhancing the quality of our article. Detailed revisions and discussions were conducted to address the reviewers' concerns. For ease of identification, we highlight all the revisions made to the manuscript in red-colored text. Meanwhile, the revised manuscript has been reviewed and edited by Language Services of Taylor＆Francis. Attached herewith is our point-by-point response to the comments raised by the reviewers.

**Responds to the reviewer’s comments:**

**Reviewer#1**:

**(1) Response to comment**: (Introduction (Lines 53-69): The introduction discusses the importance of predicting metastasis in retroperitoneal leiomyosarcoma (RLS) patients. However, it could be helpful to include a brief explanation of what retroperitoneal leiomyosarcoma is and why predicting metastasis in this particular subtype is crucial.)

**Response**:

Special thanks for your constructive comments on my manuscript. Based on your suggestions, we have provided more detailed explanations in the Introduction (Lines 49-59). The details are as follows:

Retroperitoneal leiomyosarcoma (RLS) is a relatively common histologic subtype of retroperitoneal sarcomas (RPS) that is usually incurable upon the onset of metastasis [1-3]. RLS typically manifests as a large soft-tissue mass featuring areas of necrosis located within the perirenal or posterior pararenal spaces. The most frequent growth pattern, accounting for 65% of cases, is an entirely extravascular or extraluminal mass [4]. Moreover, the mass usually exhibits a well-circumscribed margin but can appear infiltrative on occasion. En bloc resection remains the cornerstone of treatment for RLS and is the only modality with curative potential [5-7]. Of the various subtypes of PRS, RLS is particularly recalcitrant and prone to distant metastases, with 55–78% of patients developing metachronous distant metastasis (MDM) within five years post-surgery, even after undergoing R0 resection [4, 7, 8]. Metastatic RLS accounts for approximately 75% of sarcoma-related deaths, with a median survival time of just 16 months and an overall 5-year survival rate of less than 50% [4, 7].

**(2) Response to comment:** (Methods (Lines 102-114): In the "Patients" section, it would be helpful to clarify what R0 resection means for readers who may not be familiar with the term. Additionally, more information about the demographic and clinical characteristics of the patients (e.g., age, gender distribution) in both the training and validation cohorts could provide context.)

**Response**:

Thanks for your constructive suggestion, which is highly appreciated. We have revised the "Patients" section accordingly. The details are as follows:

Methods (Lines 108-110): An R0 resection was defined as the complete removal of macroscopic tumor with microscopically tumor-free resection margins [9].

Methods (Lines 116-122): Based on these criteria, a total of 121 consecutive patients who were treated at a tertiary sarcoma center between January 1, 2016, and December 31, 2021 were included in the training cohort. This cohort comprised 60 men aged 60 [49, 65] years and 61 females aged 58.2 [48, 67] years. Additionally, to assess the reliability of the model, an external validation cohort comprising 58 patients who received treatment from another tertiary sarcoma center, including 29 male and female patients aged 61 [46.5, 66] and 59 [43, 67.5] years, respectively, was established.

**(3) Response to comment:** (Methods (Lines 123-135): The "Region of Interest (ROI) Segmentation" section mentions that radiologists manually delineated the sarcoma ROIs, but it would be beneficial to provide more details on the specific criteria and guidelines used for this delineation.)

**Response**:

We sincerely appreciate the valuable comments. We made the following additions:

Supplementary Material - Appendix A3: Segmenting images into Region of Interest (ROI), such as tumors, normal tissues, and other anatomical structures, is an essential step that lays the foundation for subsequent data analysis. In the past 20 years, many popular segmentation algorithms have been applied in medical imaging research, with manual segmentation methods often used as a gold standard or ground truth. Before conducting this research, we referenced published methods for region of interest (ROI) segmentation in some radiomics studies. For example, Nie et al [10], Yang et al [11], and Wang et al [12] all used manual segmentation to delineate ROIs, and their findings revealed that the radiomics features extracted by this method were valuable for prognosis prediction; therefore, we also took a manual segmentation approach. Automatic segmentation, however, remains controversial and its accuracy requires further evaluation, as it often introduces significant variations in segmentation observed between different observers. Manual tracking boundaries also has its own inherent biases and low reproducibility of imaging experts, and is time-consuming. It is still necessary to establish a standardized, automated, and reproducible segmentation algorithm.

In addition, we added details of the manual segmentation method for ROIs in our revised manuscript (Methods, Lines 142-146): Each sarcoma ROI was manually delineated at the cross-section of the primary lesion by radiologist 1 using the ITK-SNAP software (Version 3.8, [www.itksnap.org](http://www.itksnap.org)) based on CECT images with a 5-mm thickness. Contouring was carefully performed within the borders of the tumors on arterial, venous, and delayed-phase images, avoiding covering the adjacent organs and tissues. Figure S1 and S2 show an example of the manual delineation method.

**(4) Response to comment:** (Methods (Lines 140-156): While the use of the ComBat compensation method is mentioned, a brief explanation of what this method does and why it is important for data normalization could enhance understanding.)

**Response**:

We appreciate your constructive comments. In the supplement, we briefly explain the role of the combat compensation method and its significance in terms of data normalization in the revised manuscript, as detailed below:

Methods (Lines 153-156): The Combat compensation method ([https://github.com/Jfortin1/ComBat Harmonization](https://github.com/Jfortin1/ComBat%20Harmonization)) was used to retain outperforming features in texture patterns. This method filtered the radiomic feature values affected by inconsistencies in imaging protocols, scanners, and parameters, thus improving the sensitivity of data acquired on different CT equipment [13, 14]. This method has been widely used in previous multicenter radiomics studies for radiomics feature measurement of CT and MRI images [13, 15, 16]. Then, z-scores were calculated to standardize the extracted radiomic features for all three CECT phases. Further elaboration on the role of the combat compensation method and its significance in terms of data normalization in provided in Supplementary Appendix 3.

Supplementary Material - Appendix A4: The main purpose of using ComBat compensation method is to eliminate batch effects caused by technical variations or differences in scanning devices and parameters between different image batches. These batch effects can lead to differences in feature representation between different batches of image data, which can affect the accuracy and reliability of the results. ComBat method corrects the data to make the image data from different batches more comparable and improves the interpretability of research results by preserving the biological differences between samples.

The main advantages of ComBat compensation method include:

1. Correcting batch effects: ComBat method can effectively correct the batch effects caused by technical variations or instrument differences between different batches, making the data more comparable.

2. Preserving biological differences: ComBat method corrects batch effects while trying to preserve biological differences between samples. This means that important biological information is still retained during the correction process, which helps to more accurately interpret research results.

3. Flexibility and universality: ComBat method is a general batch effect correction method that can be applied to various types of image genomics data, including gene expression data, protein expression data, metabolite data, etc. It has high flexibility and is suitable for various different research designs and data types.

In summary, ComBat compensation method plays an important role in eliminating batch effects, enhancing data comparability, and improving the interpretability of research results in image genomics. It is a commonly used and effective data analysis tool.

**(5) Response to comment:** (5. Methods (Lines 180-185): The description of follow-up assessments and distant metastasis-free survival (DMFS) could be more detailed to provide a clear understanding of how these assessments were conducted and the significance of the follow-up period.)

**Response**:

Thank you again for your valuable feedback. We hope that these additions will improve the clarity and understanding of our research findings:

Distant-metastasis-free survival (DMFS) was defined as the time from surgery to the first appearance of distant metastasis. There is no plateau of distant metastases following surgical resection of RPS, and they may still occur 5–10 years following surgery, requiring long-term close follow-up. Imaging plays a critical role in monitoring disease progression; distant metastases detected on imaging often precede clinical symptoms by months or years. Currently, there is insufficient evidence to determine the optimal follow-up interval and physical examination protocol. In patients treated with surgical resection, the National Comprehensive Cancer Network (NCCN) guidelines recommend that patients should have follow-up physical examinations and imaging (chest/abdominal/pelvic CT or MRI) every 3-6 months for 2-3 years, every 6 months for the next 2 years, and then annually thereafter. The Chinese Society of Clinical Oncology (CSCO) guidelines recommend a postoperative imaging strategy of abdominopelvic plain + enhanced CT or MRI every 3-6 months for 5 years, and then annually thereafter, as well as regular chest imaging, preferably using CT.

Synthesize the recommendations of these guidelines, the follow-up protocol in this study involved abdominopelvic plain + enhanced CT or MRI, along with chest CT every 3 months for the initial year post-surgery. After this period, the imaging frequency can be reduced to every 6 months for 2–5 years, and annually thereafter, depending on the condition of the patient and the availability of resources. Subsequent histopathologic confirmation based on core needle biopsy or surgical specimen tissue ensures the accuracy of the imaging assessment results.

We have made appropriate revisions to Methods (Lines 202-212) in the revised manuscript.

**(6) Response to comment:** (Results (Line 216): The formula used to calculate the DLR-score is provided, but some insight into what each variable in the formula represents would be useful for clarity.)

We sincerely appreciate the valuable comments. We have provided the following explanations and uploaded these additions as "Supplementary Material - Appendix A6".

The formula used to calculate the DLR-score was as follows:

*DLR-score* = 0.39669421487603296 + 0.031597 × *D_lbp-3D-k_ngtMDM_Complexity*

+ 0.066068 × *X1309* + 0.075600 × *X179* - 0.015528 × *X1312* + 0.041467 × *X1255*

+ 0.041481 × *A_original_glMDM_LargeDependenceHighGrayLevelEmphasis*

+ 0.059346 × *X1387* + 0.048740 × *X1487* - 0.091377 × *X66* + 0.050499 × *X94*

+ 0.057080 × *D_lbp-3D-m2_glszm_GrayLevelVariance* - 0.047711 × X247

- 0.062957× *V_original_glMDM_LowGrayLevelEmphasis*

- 0.048884 × *X155* - 0.034018 × *X294* + 0.028234 × *X1444*

+ 0.025147 × *D_lbp-3D-m2_glMDM_DependenceEntropy*

- 0.052596 × *X367* + 0.034525 × *X452* + 0.049526 × *X1165* - 0.054271 × *X1106*

1) The following are hand-crafted radiomics features:

*D_lbp-3D-k_ngtMDM_Complexity*

*A_original_glMDM_LargeDependenceHighGrayLevelEmphasis*

*D_lbp-3D-m2_glszm_GrayLevelVariance* - 0.047711 × X247

*V_original_glMDM_LowGrayLevelEmphasis*

*D_lbp-3D-m2_glMDM_DependenceEntropy*

In which.

1. A / D / V represents the features extracted from arterial / venous / delayed phase CECT images;

2. These five formula coefficients (0.031597, 0.041481, 0.057080, 0.062957, 0.025147) represent the weights of the hand-crafted radiomics features;

3. Radiomics features extracted by Pyradiomics

| Radiomics Feature | Characteristic |
| --- | --- |
| LowGrayLevelEmphasis | This feature is used to measure the distribution and emphasis of pixels at lower gray levels in an image. This means that the feature captures the spatial relationship between the pixels at low gray levels in an image, usually reflecting the texture characteristics of the darker areas of the image. |
| LargeDependenceHighGrayLevelEmphasis | This feature can help analyze the dependencies and emphasis between different regions of grayscale levels in an image. This means that when higher grayscale levels appear, there is a strong correlation between them, possibly representing a specific texture or structural feature. |
| Complexity | Complexity reflects the complexity of texture in an image. Typically, the higher the complexity, the more complex the texture and structure in the image, such as more variations in color, shape, size, etc. |
| GrayLevelVariance | GrayLevelariance measures the variation in gray levels between different pixels in an image. The larger the variance, the more significant the variation in gray levels, indicating a more rich and complex texture in the image, such as more color, shape, size, and other variations. Conversely, a smaller variance indicates a relatively uniform and simple texture in the image. |
| DependenceEntropy | DependenceEntropy measures the uncertainty between pixels in an image. It considers the spatial relationships between pixels and the dependencies between grayscale levels, providing information about image textures. A higher Dependence Entropy value indicates more complex and chaotic textures in the image, while a lower value indicates more uniform and regular textures. |

These features are all measurement indicators used to describe image texture features, and are widely applied in medical image analysis, especially in the areas of cancer diagnosis, recurrence and metastasis prediction, and treatment response assessment. They can provide information about tissue structure, tumor morphology, and internal heterogeneity of tissues, which can help physicians and researchers to detect abnormal regions in images, assess the severity of diseases, extract information about disease states and tissue characteristics, and provide support for clinical decision making.

Notably, combined analysis of these features provides a more comprehensive understanding of the texture features and related information in the image.

2) The following are deep learning features: *X1309, X179, X1312, X1255, X1387, X1487, X66, X94, X247, X155, X294, X1444, X367, X452, X1165, X1106.*

These deep learning features are output from the penultimate layer of the trained convolutional neural networks. I apologize for the fact that, so far, deep learning features are still not interpretable. The lack of interpretation of deep learning feature is a major obstacle to the practical application of deep learning models in clinical practice. A common approach to improving the interpretation of deep learning feature is to generate visual feature CNN activation maps and explore the decision-making implications of the attention regions [17-19]. As shown in Figure S4, the activation maps highlighted certain parts within the tumors with high predictive value in determining the MDM status. Typically, regions with high heat indicate areas of tumors that are abnormally active [17]. These regions may be characterized by features such as tumor size, shape, density, blood flow, and others. By analyzing these features, we can gain a better understanding of tumor growth and spread, thus predicting MDM status. Broadly speaking, Activation Maps of MDM tumors appear busier in comparison to Activation Maps of non-MDM tumors, which look sparser. Such visual pattern may make the process less of a “black box” and increase the interpretability of the machine diagnosis. We will endeavor to explore the interpretability of deep learning features by combining genetic and pathological information in future studies.

**(7) Response to comment:** (7. Discussion (Lines 259-268): The discussion mentions the limitations of the clinical model based on semantic imaging features and the importance of radiomics. However, it would be beneficial to elaborate on the specific limitations of relying solely on semantic imaging features and how radiomics overcomes these limitations.)

Thanks again for your suggestions. The Discussion (Lines 289-304) has been modified to elaborate on the specific limitations of relying solely on semantic imaging features and how radiomics overcomes these limitations. Details are as follows:

However, in the present study, the clinical model constructed based on the semantic imaging features identified as independent predictors of MDM in the univariate and multivariate analyses (tumor size > 10 cm) had an AUC of 0.511. This value is considerably inferior to that of the radiomics model (0.733–0.786), indicating the limited value of assessing the prognosis of RLS solely based on visual CECT features. Semantic features, such as lesion density, morphology, or size, which can be visually interpreted by radiologists, are predominantly based on empirical judgments. This approach introduces a significant degree of subjectivity in image interpretation and severely limits the consistency and diagnostic efficacy of the model.

Radiomics can overcome the limitations of semantic imaging features through image standardization processing, optimization of feature extraction algorithms, establishment of multi-modal models, enhancement of data sets, and introduction of domain knowledge, which could improve the diagnostic efficiency and consistency of the model. Moreover, the radiomics technique is characterized by automatic representative data acquisition, eliminating the need for clinical index collection, semantic feature interpretation, and manual annotation. Therefore, radiomics is readily accepted in clinical workflow. Radiomics can noninvasively capture risk-related intra- and inter-tumoral heterogeneity at the voxel level, providing a more objective and thorough means of characterizing sarcomas in a clinical setting.

**(8) Response to comment:** (8. Discussion (Lines 312-324): The discussion touches on the centralized treatment of sarcomas in dedicated sarcoma centers but does not provide details about what this entails or why it is beneficial. Including some background information on this topic would be informative for readers.)

Thank you for your valuable comments. We hope that this additional background information will be informative for readers:

Discussion (Lines 353-363): Owing to its rarity and complexity, centralized treatment of RPS in dedicated sarcoma centers entails a coordinated approach among multiple healthcare professionals. This approach can improve outcomes by ensuring that patients receive the most advanced and comprehensive treatment options. A previous nationwide study reported that the prognosis of patients with RLS may be impacted by the case volume and expertise of the treating facility [20]. Bonvalot et al. [21] reported that patients treated surgically at specialty sarcoma centers exhibited significantly better OS than those treated at non-centers, with 2-year OS of 87% vs. 70%, respectively (*P* < 0.001); notably, the multivariate analysis identified treatment at a specialized center as an independent predictor of OS, with a twofold lower odds ratio of death. Gutierrez et al. [22] also reported a higher OS for patients with retroperitoneal sarcoma treated in a dedicated sarcoma center (39 vs. 31 months, *P* = 0.011). Overall, the benefits of centralized treatment include improved coordination of care, more consistent follow-up, and access to specialized expertise and resources.

Once again, we sincerely appreciate the time and effort you put into the review process. We have tried our best to improve the manuscript and hope that the correction will meet with approval.

**References**

1.	Gamboa, A.C., A. Gronchi, and K. Cardona, Soft-tissue sarcoma in adults: An update on the current state of histiotype-specific management in an era of personalized medicine. CA Cancer J Clin, 2020. 70(3): p. 200-229.

2.	Improta, L., D. Tzanis, T. Bouhadiba, K. Abdelhafidh, and S. Bonvalot, Overview of primary adult retroperitoneal tumours. Eur J Surg Oncol, 2020. 46(9): p. 1573-1579.

3.	Ishii, T., K. Kohashi, H. Ootsuka, K. Iura, A. Maekawa, Y. Yamada, et al., Comparison between retroperitoneal leiomyosarcoma and dedifferentiated liposarcoma. Pathol Res Pract, 2017. 213(6): p. 634-638.

4.	Marko, J. and D.J. Wolfman, Retroperitoneal Leiomyosarcoma From the Radiologic Pathology Archives. Radiographics, 2018. 38(5): p. 1403-1420.

5.	Tan, M.C., M.F. Brennan, D. Kuk, N.P. Agaram, C.R. Antonescu, L.X. Qin, et al., Histology-based Classification Predicts Pattern of Recurrence and Improves Risk Stratification in Primary Retroperitoneal Sarcoma. Ann Surg, 2016. 263(3): p. 593-600.

6.	Xu, J., J. Guo, H.Q. Yang, Q.L. Ji, R.J. Song, F. Hou, et al., Preoperative contrast-enhanced CT-based radiomics nomogram for differentiating benign and malignant primary retroperitoneal tumors. Eur Radiol, 2023.

7.	Devaud, N., O. Vornicova, A.R. Abdul Razak, K. Khalili, E.G. Demicco, C. Mitric, et al., Leiomyosarcoma: Current Clinical Management and Future Horizons. Surg Oncol Clin N Am, 2022. 31(3): p. 527-546.

8.	Kannan, S., H.H. Chong, B. Chew, J.D. Ferguson, E. Galloway, T. McCulloch, et al., Leiomyosarcoma in the extremities and trunk wall: systematic review and meta-analysis of the oncological outcomes. World J Surg Oncol, 2022. 20(1): p. 124.

9.	Strauss, D.C., A.J. Hayes, K. Thway, E.C. Moskovic, C. Fisher, and J.M. Thomas, Surgical management of primary retroperitoneal sarcoma. Br J Surg, 2010. 97(5): p. 698-706.

10.	Nie, P., J. Zhang, W. Miao, S. Duan, T. Wang, J. Zhang, et al., Incremental value of radiomics-based heterogeneity to the existing risk criteria in predicting recurrence of hepatocellular carcinoma after liver transplantation. Eur Radiol, 2023. 33(9): p. 6608-6618.

11.	Yang, G., P. Nie, L. Yan, M. Zhang, Y. Wang, L. Zhao, et al., The radiomics-based tumor heterogeneity adds incremental value to the existing prognostic models for predicting outcome in localized clear cell renal cell carcinoma: a multicenter study. Eur J Nucl Med Mol Imaging, 2022. 49(8): p. 2949-2959.

12.	Wang, H., M. Zhang, J. Miao, F. Hou, Y. Chen, Y. Huang, et al., Deep learning signature based on multiphase enhanced CT for bladder cancer recurrence prediction: a multi-center study. EClinicalMedicine, 2023. 66: p. 102352.

13.	Orlhac, F., F. Frouin, C. Nioche, N. Ayache, and I. Buvat, Validation of A Method to Compensate Multicenter Effects Affecting CT Radiomics. Radiology, 2019. 291(1): p. 53-59.

14.	Orlhac, F., A. Lecler, J. Savatovski, J. Goya-Outi, C. Nioche, F. Charbonneau, et al., How can we combat multicenter variability in MR radiomics? Validation of a correction procedure. Eur Radiol, 2021. 31(4): p. 2272-2280.

15.	Liu, S., W. Sun, S. Yang, L. Duan, C. Huang, J. Xu, et al., Deep learning radiomic nomogram to predict recurrence in soft tissue sarcoma: a multi-institutional study. Eur Radiol, 2022. 32(2): p. 793-805.

16.	Wang, T., J. Hao, A. Gao, P. Zhang, H. Wang, P. Nie, et al., An MRI-Based Radiomics Nomogram to Assess Recurrence Risk in Sinonasal Malignant Tumors. J Magn Reson Imaging, 2023. 58(2): p. 520-531.

17.	Song, H., S. Yang, B. Yu, N. Li, Y. Huang, R. Sun, et al., CT-based deep learning radiomics nomogram for the prediction of pathological grade in bladder cancer: a multicenter study. Cancer Imaging, 2023. 23(1): p. 89.

18.	Mazin, A., S.H. Hawkins, O. Stringfield, J. Dhillon, B.J. Manley, D.K. Jeong, et al., Identification of sarcomatoid differentiation in renal cell carcinoma by machine learning on multiparametric MRI. Sci Rep, 2021. 11(1): p. 3785.

19.	Kundu, R., P.K. Singh, S. Mirjalili, and R. Sarkar, COVID-19 detection from lung CT-Scans using a fuzzy integral-based CNN ensemble. Comput Biol Med, 2021. 138: p. 104895.

20.	Keung, E.Z., Y.J. Chiang, J.N. Cormier, K.E. Torres, K.K. Hunt, B.W. Feig, et al., Treatment at low-volume hospitals is associated with reduced short-term and long-term outcomes for patients with retroperitoneal sarcoma. Cancer, 2018. 124(23): p. 4495-4503.

21.	Bonvalot, S., E. Gaignard, E. Stoeckle, P. Meeus, G. Decanter, S. Carrere, et al., Survival Benefit of the Surgical Management of Retroperitoneal Sarcoma in a Reference Center: A Nationwide Study of the French Sarcoma Group from the NetSarc Database. Ann Surg Oncol, 2019. 26(7): p. 2286-2293.

22.	Gutierrez, J.C., E.A. Perez, F.L. Moffat, A.S. Livingstone, D. Franceschi, and L.G. Koniaris, Should soft tissue sarcomas be treated at high-volume centers? An analysis of 4205 patients. Ann Surg, 2007. 245(6): p. 952-8.

23.	Landis, J.R. and G.G. Koch, The measurement of observer agreement for categorical data. Biometrics, 1977. 33(1): p. 159-74.

**Reviewer#2**:

**(1) Response to comment**: (1. Please provide an explanation of the construction process and usage parameters for the clinical model, RS model, and DLR model mentioned in the article.)

**Response**:

Thanks for your constructive suggestion, which is highly appreciated. We have made the following revisions and supplements in the revised manuscript:

Methods (Lines 182-185): The clinical model was built upon independent clinicoradiological factors predicting MDM identified through univariate and multivariate logistic regression analyses (tumor size > 10 cm, as shown in Table 2).

Supplementary Material - Appendix A5: For RS model constructed, the hand-crafted features were extracted from the ROIs of three-phase CECT images using an open-source Python package (Pyradiomics), including (1) intensity statistic features, including features that quantitatively delineate the distribution of voxel intensities within the ROIs through commonly used and basic metrics; (2) shape features, including 3-D features, which reflect the shape and size of the ROIs; (3) texture features, which are composed of features calculated by gray level co-occurrence matrix (GLCM), gray level run length matrix (GLRLM), gray level size zone matrix (GLSZM), gray level dependence matrix (GLDM), and neighboring gray tone difference matrix (NGTDM) quantifying the heterogeneity differences of ROIs; and (4) filter and wavelet features, which include the intensity and texture features derived from filter transformation and wavelet transformation of the original images, obtained by applying filters such exponential, logarithm, square, square root, gradient, lbp_2D, lbp_3D_k and wavelet (wavelet-LHL, wavelet-LHH, wavelet-HLL, wavelet-LLH, wavelet-HLH, wavelet-HHH, wavelet-HHL and wavelet-LLL). The instructions of theradiomics features are shown in Pyradiomics document (Version 3.0) on <https://pyradiomics.readthedocs.io>.

Results (Lines 231-233): Overall, 5,329 hand-crafted features from the three-phase CECT images exhibited high reproducibility (ICC > 0.8); in the screening using the MRMR algorithm and LASSO logistic regression (Figures S3a-b), 24 most valuable hand-crafted features (Figures S3c) were selected to develop the RS model.

Supplementary Material - Appendix A5: For deep learning analysis, the CECT images were set to soft tissue window (W:350, L:35). The largest cross section was derived from the 3D-ROI, and this ROI was extended outward into a square area. The cropped area was resized to 224×224 pixels as the input channels of ResNet18. The pre-trained ResNet18 model in the Pytorch framework was implemented. DL features were extracted from the largest cross-section of the 3D-ROI, by the pre-trained classification model ResNet18. In the ResNet-18 model, the output of the penultimate layer of the trained CNN was used to define the deep learning features; after eliminating the null features, 1,536 deep learning features were ultimately extracted from the ROIs of the three-phase CECT images.

Results (Lines 233-237): For developing the DLRS model, 1,536 deep learning features were combined with 5,329 hand-crafted radiomics features for inclusion in the subsequent analyses; by using the MRMR algorithm, 25 radiomics/DL features were selected and entered into the LASSO logistic regression model (Figures 3a-b); finally, 16 deep learning features and five hand-crafted features (Figures 3c) were combined to construct the DLRS.

**(2) Response to comment:** (2. Were the "semantic features" extracted from medical images collected by professional radiologists? Were they subjected to consistency evaluation, repeat measurement, and other steps to ensure their reliability?)

**Response**:

Thanks for your constructive suggestion, which is highly appreciated. We apologize for the omission of this detail, which may confuse readers. First, the "semantic features" were all collected by professional radiologists; furthermore, they were assessed for consistency. For clarity, we have made the following supplements in the revised manuscript:

Methods (Lines 131-136): All CECT images were reviewed independently by two professional radiologists with more than 15 years experience in abdominal imaging diagnosis. Imaging semantic features included tumor size, CT-reported LN status, cystic spaces or necrosis, degree of enhancement, enhancement pattern, tumor contours, and adjacent organ involvement. Cohen’s Kappa coefficient and intraclass correlation coefficient (ICC) were used to evaluate inter-observer agreement for semantic features [23]. Generally, the Kappa coefficient/ICC of 0–0.20 indicates a poor agreement; 0.21–0.40 fair agreement; 0.41–0.60 moderate agreement; 0.61–0.80 good agreement, > 0.80 excellent agreement [23]. In this study, the Cohen’s Kappa coefficients/ICCs for semantic features showed good agreement between the radiologists as detailed in Table S1.

**Table S1.** The interobserver agreement of CECT semantic features for retroperitoneal leiomyosarcoma

| CECT semantic feature | Disagreement | Kappa value/ICC | 95% CI |
| --- | --- | --- | --- |
| Tumor size | 10 (5.6) | 0.858 | 0.806-0.908 |
| CECT-reported LN status | 4 (2.2) | 0.937 | 0.902-0.976 |
| Cystic spaces or necrosis | NA | 0.985 | 0.975-0.990 |
| Degree of enhancement | 9 (5.0) | 0.846 | 0.801-0.892 |
| Enhancement pattern | 7 (3.9) | 0.863 | 0.826-0.900 |
| Tumor contours | 3 (1.7) | 0.947 | 0.922-0.972 |
| Adjacent organ involvement | NA | 0.989 | 0.979-0.993 |

Abbreviations: CECT, contrast-enhanced computed tomography; CI, confidence interval. ICC, intraclass correlation coefficient.

**(3) Response to comment**: (3.What diagnostic methods were used to confirm the presence of MDM in the patients in this study? Were they considered the gold standard?)

**Response**:

We sincerely appreciate the valuable comments. We have added the following details in the Methods (Lines 205-212):

Imaging plays a critical role in monitoring disease progression; DM detected on imaging often precede clinical symptoms by months or years. As recommended by the Chinese Society of Clinical Oncology (CSCO) guidelines and National Comprehensive Cancer Network (NCCN) guidelines, the follow-up protocol in this study involved abdominopelvic plain + enhanced CT or MRI, along with chest CT every 3 months for the initial year post-surgery. After this period, the imaging frequency can be reduced to every 6 months for 2–5 years, and annually thereafter, depending on the condition of the patient and the availability of resources. Subsequent histopathologic confirmation based on core needle biopsy or surgical specimen tissue ensures the accuracy of the imaging assessment results.

**(4) Response to comment**: (4. Did all patients in this study receive adjuvant therapy after surgery? Please provide an explanation.)

**Response**:

Thanks for your suggestion. The specifics are as follows:

In this study, all patients included did not undergo preoperative neoadjuvant therapy. Currently, there is no consensus on the application of adjuvant chemotherapy or radiotherapy in the treatment of RLS, and there is also a lack of high-quality research to guide decision-making. Thus, adjuvant therapy is not a standard practice in our centers; instead, it is reserved for a select few patients based on individualized considerations following multidisciplinary team discussions. Data pertaining to adjuvant therapy were not gathered in this study, and was not factored into the outcome analysis.

**(5) Response to comment**: (5. The KI67 index was included as a clinical feature. Would including invasive medical features in the model create ambiguity regarding the emphasis on "preoperative" in this article?)

**Response**:

Thanks again for your comments. We apologize for the ambiguity caused by our absence of description.

In this study, the immunohistochemistry findings of Ki67 were obtained from preoperative sarcoma biopsies (Methods, Lines 185-186). According to the Chinese Society of Clinical Oncology (CSCO) guidelines, image-guided core needle biopsy is recommended before treatment for retroperitoneal sarcoma (RPS) except for high-grade liposarcoma. A definitive pathologic diagnosis from the biopsy helps the Multidisciplinary Team (MDT) in developing a rational surgical protocol and a comprehensive treatment plan. Moreover, biopsies under image guidance are strongly advised in specific circumstances such as:

1. To differentiate metastatic tumors, lymphomas, germ cell tumors, etc.;

2. When imaging evaluation of the tumor is difficult to resect;

3. When there is distant metastasis;

4. Prior to preoperative neoadjuvant therapy.

**(6) Response to comment**: (6.On line 84, it is stated that " One means of improving prognosis could involve the use of radiomics, which enables the extraction of mineable high-throughput quantitative features from conventional medical images". Radiomics is a method of extracting a large number of quantitative features from medical images and using these features to predict a certain state and assist doctors in making decisions. It is often used in disease prognosis evaluation, treatment response prediction, and other areas, but it is not a means of improving disease prognosis.)

**Response**:

Thanks very much for your careful scrutiny and suggestions. We apologize for our misrepresentation and have revised the original sentence to read as follows:

Introduction (Lines 88-91): Radiomics offers a valuable approach for assessing tumor prognosis by enabling the extraction of mineable high-throughput quantitative features from medical images. This approach facilitates the capture of tissue features and lesion characteristics, such as the heterogeneity and shape of tumors, that are not discernible to the naked eye of radiologists.

**(7) Response to comment**: (7. In the discussion section, it is stated that " As shown in Figure S4, the CNN activation maps highlighted certain parts of the sarcomas with high predictive value in determining the MDM status. " Please provide a specific explanation of Figure S4. What are the imaging characteristics of the high-heat areas, and why do they have predictive value for MDM status?)

**Response**:

Thanks again for your suggestion. We have rewritten the title of Figure S4 and provided a detailed explanation of the imaging characteristics of the high-heat areas in the discussion (lines 336-345) as detailed below:

Title: Figure S4 presents the Activation Maps showing important retroperitoneal leiomyosarcoma regions that the deep convolutional neural networks learned as the most predictive of distant metastasis. The highlighted areas represent the regions of high value in the model’s distant metastasis prediction, whereas the suppressed areas are regions with little important predictive value for distant metastasis.

Discussion (Lines 336-345): The lack of interpretation of deep learning feature is a major obstacle to the practical application of deep learning models in clinical practice. A common approach to improving the interpretation of deep learning feature is to generate visual feature CNN Activation Maps and explore the decision-making implications of the attention regions [17-19]. As shown in Figure S4, the Activation Maps highlighted certain parts within the tumors with high predictive value in determining the MDM status. Typically, regions with high heat indicate areas of tumors that are abnormally active [17]. These regions may be characterized by features such as tumor size, shape, density, blood flow, and others. By analyzing these features, we can gain a better understanding of tumor growth and spread, thus predicting MDM status. Broadly speaking, Activation Maps of MDM tumors appear busier in comparison to those of non-MDM tumors, which look sparser. Such visual pattern may make the process less of a “black box” and increase the interpretability of the machine diagnosis. We will endeavor to explore the interpretability of deep learning features by combining genetic and pathological information in future studies.

Once again, we sincerely appreciate the time and effort you put into the review process. We have tried our best to improve the manuscript and hope that the correction will meet with approval.

**References**

17.	Song, H., S. Yang, B. Yu, N. Li, Y. Huang, R. Sun, et al., CT-based deep learning radiomics nomogram for the prediction of pathological grade in bladder cancer: a multicenter study. Cancer Imaging, 2023. 23(1): p. 89.

18.	Mazin, A., S.H. Hawkins, O. Stringfield, J. Dhillon, B.J. Manley, D.K. Jeong, et al., Identification of sarcomatoid differentiation in renal cell carcinoma by machine learning on multiparametric MRI. Sci Rep, 2021. 11(1): p. 3785.

19.	Kundu, R., P.K. Singh, S. Mirjalili, and R. Sarkar, COVID-19 detection from lung CT-Scans using a fuzzy integral-based CNN ensemble. Comput Biol Med, 2021. 138: p. 104895.

23.	Landis, J.R. and G.G. Koch, The measurement of observer agreement for categorical data. Biometrics, 1977. 33(1): p. 159-74.

We sincerely appreciate the editor/reviewers' warm work and hope that the corrections will meet with approval. Thank you again for your consideration. We look forward to hearing from you.

Sincerely,

[Corresponding author: Daorong Wang]

[Affiliation: Northern Jiangsu People's Hospital, Clinical Teaching Hospital of Medical School, Nanjing University, Yangzhou, China]

[Postal address: No.98 Nantong West Road, Yangzhou, Jiangsu Province, China]

[Email address: [wdaorong666@sina.com](mailto:wdaorong666@sina.com)]
